# Preparation of graphene oxide-doped silica aerogel using supercritical method for efficient removal of emerging pollutants from wastewater

**DOI:** 10.1038/s41598-023-43613-w

**Published:** 2023-09-30

**Authors:** Subhash Kumar Sharma, P. Ranjani, Hadas Mamane, Rajnish Kumar

**Affiliations:** 1 Department of Chemical Engineering, Indian Institute of Technology Madras, Chennai, 600036 India; 2 School of Mechanical Engineering, Faculty of Engineering, Tel Aviv University, 69978 Tel Aviv, Israel

**Keywords:** Pollution remediation, Sustainability, Chemical engineering, Synthesis of graphene

## Abstract

Emerging pollutants and a large volume of unused dyes from the textile industry have been contaminating water bodies. This work introduces a scalable approach to purifying water by the adsorption of Acid green 25 (AG), Crystal Violet (CV), and Sulfamethoxazole (SMA) from an aqueous solution by graphene oxide (GO) doped modified silica aerogel (GO-SA) with supercritical fluid deposition (SFD) method. Characterization of GO-SA using X-ray diffraction (XRD), Fourier-transform infrared spectroscopy (FTIR), high-resolution scanning electron microscopy (HR-SEM), thermogravimetric analysis (TGA), and Brunauer–Emmett–Teller (BET) adsorption isotherms revealed the improvement in the adsorbent surface area, and its textural properties. The high removal percentages observed in most of the experimental runs provide evidence of the excellent performance of the adsorbent towards the anionic and cationic dyes along with the antibiotic. The adsorption isotherm and kinetics showed that the Langmuir isotherm and pseudo-second-order kinetic models could explain adsorption. The adsorbent holds a higher adsorption capacity for SMA (67.07 mg g^−1^) than for CV (41.46 mg g^−1^) and AG (20.56 mg g^−1^) due to the higher hydrophobicity that interacts with the hydrophobic adsorbent. The GO-SA successfully removed AG, CV, and SMA with removal percentages of 98.23%, 98.71%, and 94.46%, respectively. The parameters were optimized using Central Composite Design (RSM-CCD). The prepared aerogel showed excellent reusability with a removal efficiency of > 85% even after 5 cycles. This study shows the potential of GO-SA adsorbent in textile and other wastewater purification.

## Introduction

India has only 4% of the global water resources, which mainly comes from lakes, rivers, and ponds, while it supports 18% of the world’s population. Moreover, as an agrarian country, India is undergoing massive urbanization, with the urban population increasing from 28% in 2000 to 34.9% in 2020^1^. Despite an average GDP growth rate of 6% and increasing life expectancy, India's pharmaceuticals, automotive, chemical, petrochemical, and other manufacturing industries are facing severe water scarcity^2,3^. Emerging contaminants in the water, such as industrial chemicals, textiles, pharmaceuticals, hospital waste, and pesticides, pose a significant hazard to water quality^4–6^. These contaminants harm the ecosystem, and even trace amounts of specific chemicals can have a substantial impact^7–9^.

The increase in population and urbanization has led to a rapid demand for synthetic dyes and an expansion of the textile industry^10^. It is estimated that worldwide, 7 × 10^5^–1 × 10^6^ tons^11,12^ of dyes are produced annually and used in various industries such as textiles, plastics, dyeing, paper, pulp, color photographs, cosmetics, and other industrial products^13–16^. However, most of these synthetic dyes^17^ are toxic, and the wastewater from this industry must be treated before discharge into water bodies to reduce water pollution and preserve living organisms^18^. Discharge of this contaminated water poses a significant environmental concern^19^. Around 40–50% of dyes are discharged as waste during the dying process, and approximately 15–20% are rejected as wastewater, which changes the color of water and generates foam on the surface of water bodies. This is another major threat to the environment^20^. Moreover, most artificial dyes are known to be endocrine disruptors, mutagenic, and carcinogenic, which can affect the health of a wide range of organisms.

The accumulation of organic dyes in water bodies can also hinder light penetration, which can impede the natural process of decontamination and photosynthesis^21^. Various treatment methods and combinations are used to remove organic dyes from water bodies, such as membrane filtration, coagulation, flocculation, ozonation, advanced oxidation, and biological processes. However, some of these methods face significant challenges, such as high capital costs and large volumes of sludge generation^22^.

Dyes are not the only contaminants in wastewater streams that end up in natural water bodies^23^; pharmaceutical molecules are also major contributors to water pollution. Common drug molecules, such as antibiotics, non-steroidal anti-inflammatory drugs, fat/lipid regulators, hormones, and psychotropics, are excreted by humans and animals and ultimately make their way into water bodies^5,24^. For example, drugs used to various infections are consumed in large quantities and can be detected in water bodies. Another concerning factor is antimicrobial resistance, exacerbated by heavy use of antibiotics in humans and livestock^25^. Sulfamethoxazole, a common antibiotic-derived compound, is often found in various water bodies^26^. Unfortunately, existing conventional wastewater treatment methods are mainly incapable of removing the trace amounts of pharmaceuticals in water^27^. To avoid further contamination of natural water bodies, alternative treatment and separation methods are required^28^. Various methods, such as advanced oxidation processes (AOPs), adsorption, and membrane filtration, are being developed and tested to remove persistent antibiotics^29^.

Aerogels can be prepared from a variety of sources, including inorganic materials (such as zirconia, titania, alumina, and silica), artificial polymers^30^ (like PVC and formaldehyde), carbon-based materials^31^ (such as carbon and graphene), polyimide, polystyrene, and polyurethane^32^. The surface of these aerogels can be modified using supercritical deposition (SFD), which involves the use of supercritical fluids maintained at a temperature and pressure beyond their critical point. SFD has several advantages over conventional deposition techniques, including depositing materials at a lower temperature, reducing environmental impact, and improving film quality^33,34^. As a result, SFD has found applications in various fields, such as the production of microelectronics, biomedical implants, and energy storage devices^35^.

Graphene-doped modified silica aerogels (GO-SA) typically have a high surface area due to their porous and lightweight nature. This high surface area allows for a greater number of active sites available for adsorption, making them more efficient at removing contaminants from water. The unique structure of GO-SA, with graphene sheets incorporated into the silica matrix, enhances their adsorption capacity^36^. Graphene's two-dimensional structure and high surface area provide additional binding sites for contaminants, leading to improved adsorption performance^37–41^. GO-SA often exhibit rapid adsorption kinetics, meaning they can quickly adsorb contaminants from water^42,43^. This can be crucial when fast removal of pollutants is required, such as in emergency water treatment or environmental cleanup efforts. GO-SA can be tailored to target specific contaminants by modifying their surface chemistry^44^. Functionalization of the aerogel surface can enhance its selectivity for pollutants, making it a versatile option for addressing various water quality issues. GO-SA can be regenerated and reused multiple times, reducing waste and operational costs. Regeneration methods typically involve desorbing the adsorbed contaminants from the aerogel, restoring its adsorption capacity^45,46^.

Compared to some other adsorbents, GO-SA can be considered environmentally friendly^47,48^. They are often produced using eco-friendly sol–gel methods and can be synthesized with relatively low energy consumption. Additionally, their reusability reduces the need for frequent disposal. GO-SA can exhibit resistance to fouling, meaning they can continue to adsorb contaminants effectively even when exposed to complex water matrices with high levels of organic matter or other interfering substances^36^. The synthesis of GO-SA can be scaled up for larger water purification applications. This scalability makes them suitable for both small-scale and large-scale water purification projects.

This study doped a modified superhydrophobic aerogel with graphene oxide (GO) using SFD to remove sulfamethoxazole (SMA), acid green 25, and crystal violet. These water-soluble contaminants often end up in domestic wastewater and are major contributors to water pollution^49,50^. The aerogel was characterized using various techniques, such as XRD, FTIR, SEM, TGA, and BET, to better understand its properties before and after use. The effect of several parameters, including the adsorbent dosage, contact time, pH of the solution, and initial dye concentration, was determined for removal efficiency. To optimize the experimental system, response surface methodology (RSM) constructed on central composite designs (CCD) was used as an operative statistical and mathematical method to identify the effectiveness of different parameters concurrently assessed^22,39,41,49,51^. Process optimization was then used to determine the optimal combination of input variables that result in the maximum or minimum response. The novelty of this study lies in its innovative approach to combatting water pollution caused by an emerging pollutant from the textile industry and pharmaceutical waste. By introducing a scalable purification method involving the use of (GO-SA) with a supercritical deposition method, this study has harnessed the unique properties of this modified material to achieve remarkable contaminant removal efficiencies. The synergistic effects of graphene oxide and silica aerogel in the adsorption process have not only significantly enhanced the adsorption capacity. The exceptional reusability of the GO-SA adsorbent further underscores its practicality and sustainability for wastewater purification. This study presents a cutting-edge and highly effective solution with broad implications for addressing complex water pollution challenges in various industrial and environmental contexts.

## Experimental

### Materials

Indogas supplied CO_2_ (purity > 99.999%). The dyes, Acid green 25, Crystal violet, graphite powder and monopotassium phosphate, were sourced from Sigma-Aldrich. Silica, [(trimethylsilyl)oxy]-modified aerogel was sourced from Cabot (Riga LV-1039, Latvia), Sulfamethoxazole (SMA) 98% were sourced from TCI Japan. Merck supplied acetic acid. Absolute ethanol was supplied by Honeywell, and Thermo Fisher Scientific supplied absolute methanol. Acetonitrile and acetone, the analytical grade, were provided by Spectrochem. All reagents were used without further treatment/purification. The aerogel surface was modified using graphene oxide by the supercritical deposition technique. The characteristics of used contaminants can be seen in Table S1 of Supplementary information.

### Supercritical high-pressure reactor assembly for GO doping

**Figure 1:**
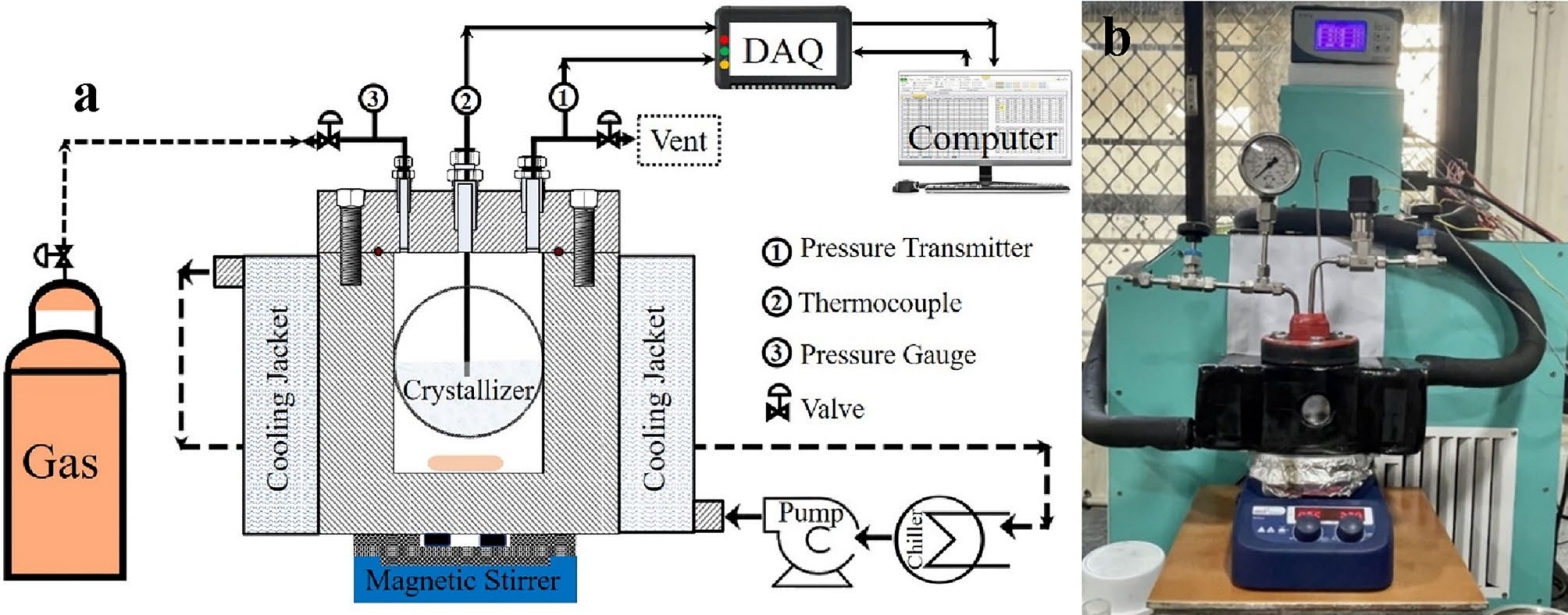
(**a**) Schematic experimental setup for the high-pressure supercritical CO_2_ reactor (**b**) Real time picture of the experimental setup for the high-pressure supercritical CO_2_ reactor.

Custom-made high-pressure SS-316 reactors were used to dope GO using supercritical method, as shown in Fig. 1. The reactor is equipped with circular quartz windows for visual observation, a magnetic stirrer at the bottom of the reactor for mixing its contents, and a cooling jacket that circulates an ethylene glycol/water mixture from an external refrigerated and heating circulator (Siskin Profichill RCC1200-ST40) to regulate the temperature of the reactor. Pressure inside the reactor was measured with a Baumer pressure transducer, and an analog pressure gauge, while temperature was measured with a RTD thermocouple. Both instruments were connected to a Data Acquisition (DAQ) system (Make: PPI, Mumbai, India) which was connected to a computer that recorded and displayed the pressure and temperature readings every second, using “ProLog” software.

### GO doping over modified silica aerogel using supercritical CO_2_

Graphene oxide (GO) was prepared using a modified Hummer’s method from graphite powder, as Qana et al.^52^ described. In a typical experiment, 100 mg of GO was dissolved in 10 ml of ethanol and placed in a reactor with a magnetic stirrer. Approximately 1 g of modified silica aerogel was placed in a mesh bucket and added to the reactor to avoid direct contact with GO. The reactor was sealed and filled with CO_2_ at 10 °C, reaching a pressure of 6 MPa. The temperature was then raised to 80 °C, increasing the pressure to 15 MPa (which is possible due to the solvent being in supercritical phase), and maintained under stirring for 6 h. After 6 h, the reactor was slowly depressurized (45 min). The obtained GO-doped modified silica aerogel was washed with ethanol and air-dried at 60 °C for 12 h.

### Contaminant adsorption onto GO-SA Aerogel

GO-doped modified silica aerogel was activated by placing 1 g of the granular aerogel in a separating funnel, to which 10 mL of pure ethanol was added^37^. After eluting the ethanol, 20 mL of Milli-Q water was added to rinse the activated aerogel to eliminate remaining ethanol. Thereafter, this activated aerogel was denoted as GO-SA. The adsorption of contaminants was examined using GO-SA, and the percentage of contaminants removed was calculated using Eq. (1).

1 $$Removal \left(\%\right)=\frac{{c}_{0}-{c}_{e}}{{c}_{e}} \times 100$$

### Batch experiment

**Figure 2:**
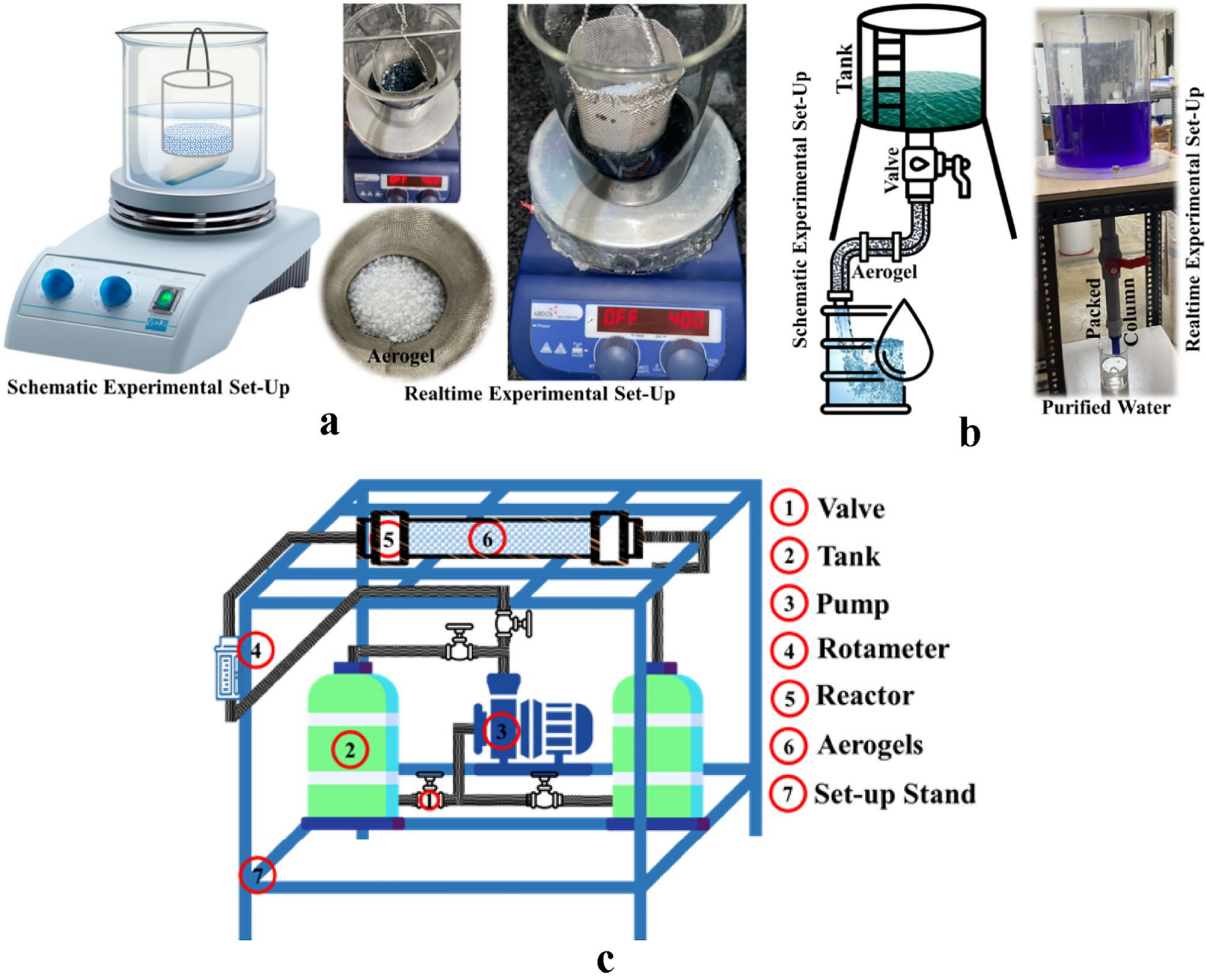
Experimental setup (**a**) batch experiments (**b**) semi-batch experiments with aerogel packed column and controlled flow due to gravity (**c**) continuous reactor packed with aerogels.

All the water purification studies were carried out in a 250 mL beaker filled with 100 mL of solution, as shown in Fig. 2a for batch studies. Solutions containing different contaminants were prepared in Milli-Q water at the required concentration to fit the RSM design. Batch adsorption studies were conducted in a flask and a beaker with a magnetic stirrer, using a pre-set quantity of adsorbent and a desired concentration of contaminants. For kinetic studies, the aerogel dose was 12.5 g L^−1^ with a contaminant concentration of 70 mg L^−1^. Sampling was done at a regular interval (0, 2, 4, 6, 8, 10, 13, 16, 19, 22, 25, 30, 35, and 40 min) to measure the concentrations of contaminants in the mother liquor. To study the isotherm, initial concentration of the contaminant solution was varied from 10 to 100 mg L^−1^. The removal efficiency was further tested in a semi-batch scale study, as shown in Fig. 2b, with a controlled flow rate through a packed column of aerogel. A continuous process for removing emerging contaminants and an easily scalable process using an aerogel-packed reactor were studied using an experimental setup shown in Fig. 2c.

### Contaminants quantification

**Figure 3:**
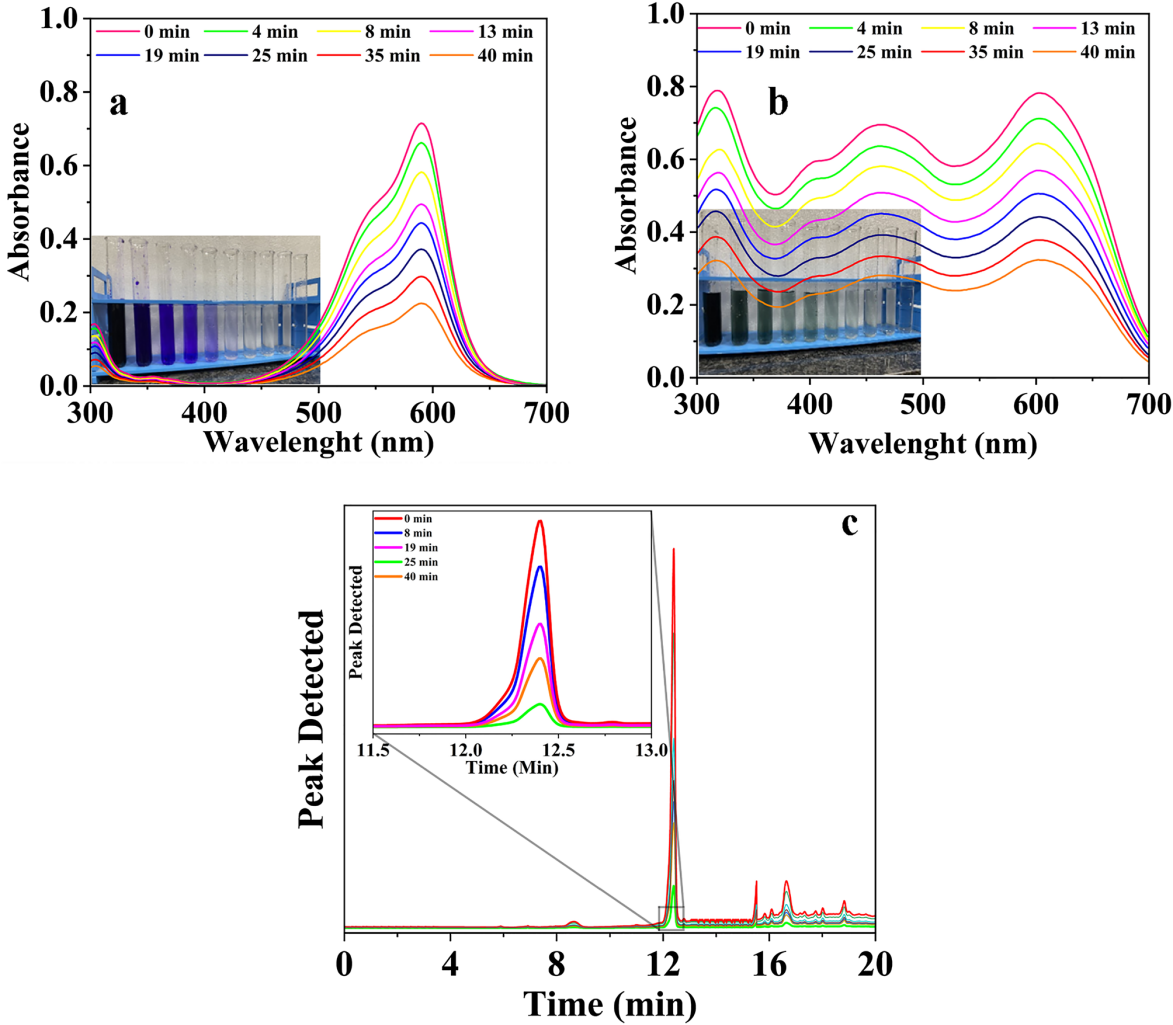
(**a**) UV–Vis spectra of CV aqueous solutions adsorbed by GO-SA, (**b**) UV–Vis spectra of AG aqueous solutions adsorbed by GO-SA, (**c**) HPLC chromatogram of SMA aqueous solutions adsorbed by GO-SA.

The dyes used in this study, acid green (AG) and crystal violet (CV), were quantitatively analyzed using UV–Vis Spectroscopy (Jasco Model–V 730). the UV–Vis spectra of CV and AG aqueous solution adsorbed by GO-SA shown in Fig. 3a and b. The pharmaceutical contaminants sulfamethoxazole (SMA) were quantified using an HPLC (Shimadzu SPD-M20A) equipped with a HiQ Sil C18HS column (250 × 4.6 mm) and a PDA-UV detector. A mobile phase of phosphate buffer (A), and acetonitrile (B) were used for the HPLC analysis. In a typical analysis, the mobile phase consisting of 90% (A) and 10% (B) was allowed to flow for 1.5 min, with a flow rate of 0.8 mL min^-1^. A gradient over 12 min was then set for the mobile phase composition of 5% (A) and 95% (B), which was held for 10 min. The PDA-UV absorbance was set to 254 nm for quantification purposes, the HPLC chromatogram of SMA aqueous solution adsorbed by GO-SA is shown in Fig. 3c. All experiments were conducted in triplicate to ensure experimental error was minimized.

### GO-SA regeneration and reusability

Experimental conditions for the reusability study were the same as those for the kinetics study for all contaminants. The GO-SA adsorbent was regenerated after each experiment by washing the used aerogel with different eluents and ethanol. This process was repeated three times, followed by three water washes, to ensure complete removal of all adsorbed contaminants. The aerogel was then dried at 60 °C for 4 h. The regenerated, and dried aerogel was used again in subsequent experiments to study its reusability efficiency. Each aerogel sample was reused five times to determine the decrease in separation efficiency.

### Adsorption isotherms, kinetics, and thermodynamics studies

Initially, the effects of pH, contact time, adsorbent dose, and contaminant concentration were studied using the RSM experimental design. These variables were varied over the ranges of pH 4–9, contact time 15–40 min, adsorbent dosage 5–12.5 g L^−1^, and contamination concentration 25–70 mg L^−1^. The experiments were conducted in 100 mL batches, with doses ranging from 0.5 to 1.25 g depending on the specific conditions. After determining the optimized factors through the RSM designed experiments, kinetics and isotherms studies were conducted using the maximum contaminant concentration, contact time, adsorbent dosage, and optimum pH.

To conduct the kinetics study, 1.25 g of GO-SA was added to 70 ppm of contaminants (pH 6.5–7) in an orbital shaker at 90–100 rpm and room temperature to minimize mass transfer resistances and achieve equilibrium more quickly. Samples were taken at regular intervals of 2 min for quantification by UV–Vis and HPLC. To conduct the isotherms study, 1.25 g of GO-SA was added to a flask containing 70 ppm of contaminants and placed in an orbital shaker for 40 min at 90–100 rpm and room temperature. The concentration of contaminants adsorbed on the aerogel was determined using Eq. (2).

2 $${Q}_{e}=\frac{{C}_{0}-{C}_{e}}{m}\times v$$

At equilibrium, the quantity of contaminants adsorbed on the adsorbent is represented by *Q*_*e*_ (mg L^−1^). The contaminant's initial concentration in the solution is *C*_*0*_ (mg L^−1^), and its concentration after adsorption is *C*_*e*_ (mg L^−1^). The amount of adsorbent used is *m* (g), and the volume of the solution is *v* (L). Different isotherms, such as Langmuir adsorption, Freundlich adsorption, pseudo-first-order adsorption, and pseudo-second-order adsorption, were used to determine the best fit and evaluate the adsorption pattern of the contaminants on GO-SA.

The Langmuir adsorption isotherm is based on several assumptions: (i) the surface of the adsorbent is homogeneous, meaning that all binding positions are practically equal^53–55^. (ii) adsorbed molecules do not interact with one another, (iii) the adsorption process is the same throughout the experimental time scale, and (iv) at the maximum adsorption, only a monolayer of contaminations could be seen on the surface. The Langmuir isotherm is written as Eq. (3):

3 $$\frac{1}{{q}_{e}}=\frac{1}{{c}_{e}{q}_{m}{k}_{L}}+\frac{1}{{q}_{m}}$$

where $${C}_{e}$$ (mg L^−1^) is contaminants concentration at equilibrium, $${q}_{e}$$ (mg g^−1^) are contaminants adsorbed on GO-SA at equilibrium, $${q}_{m}$$ (mg g^−1^) are maximum contaminants adsorbed on GO-SA, $${k}_{L}$$ (L mg^−1^) is Langmuir constant, and $${C}_{0}$$ initial concentration. The Langmuir isotherm is articulated with a relation called separation factor *R*_*L*_ written as Eq. (4), which suggests the nature of adsorption whether it is favorable ($$0{<R}_{L}<1)$$, irreversible ($${R}_{L}=0$$) and unfavorable ($${R}_{L}=1)$$

4 $${R}_{L}=\frac{1}{1+{k}_{L}{c}_{0}}$$

Freundlich adsorption isotherm applies to both multilayer and heterogeneous molecule adsorption. It gives an understanding that designates surface heterogeneity as well as the exponential function of the active site and its binding energy^32,36,56^. Freundlich isotherm is written as Eq. (5):

5 $$\mathit{log}{q}_{m}=\mathit{log}{k}_{F}+\frac{1}{n}\mathit{log}{c}_{e}$$

where ${k}_{F}$ is isotherm constant, and $n$ is the Freundlich constant interrelated to the promoted degree of adsorption. When the value of $n$ is between 1 and 10, the adsorption is advantageous.

Temkin adsorption isotherm relates the adsorbate and adsorbent, which overlooks the very large and low concentration value. This model adopts the temperature as a function in which the layer of molecules decreases linearly compared to logarithmic^54,57^. The Temkin isotherm can be written as Eq. (6):

6 $${q}_{e}=\frac{RT}{{b}_{t}}\mathit{ln}{A}_{T}+\frac{RT}{{b}_{t}}\mathit{ln}{C}_{e}$$

where ${A}_{T}$ is equilibrium Temkin isotherm constant, ${b}_{t}$ is the Temkin isotherm constant, and T temperature.

Pseudo-first-order kinetic model is built on the hypothesis that the rate of variation of contaminant uptake with time is proportional to the difference in saturation concentration and the amount of contaminant uptake with time, which is commonly relevant over the initial stage of an adsorption process^58^. It is written as Eq. (7):

7 $$\mathit{log}\left({q}_{e}-{q}_{t}\right)=\mathit{log}{q}_{e}-\frac{{k}_{1}}{2\cdot 303}t$$

where ${q}_{e}$ and ${q}_{t}$ are the contaminants adsorbed per mass of adsorbent (mg g^−1^) at equilibrium and any time, respectively, and ${k}_{1}$ is the rate constant of first-order adsorption.

The pseudo-second-order kinetic model predicts behavior across the spectrum of adsorption and is based on the premise that the rate-limiting phase is chemisorption. In this case, the adsorption rate is determined by adsorption capacity rather than adsorbate concentration^38,59^. It is written as Eq. (8):

8 $$\frac{t}{{q}_{t}}=\frac{1}{{k}_{2}{q}_{e}^{2}}+\left(\frac{1}{{q}_{e}}\right)t$$

where ${k}_{2}$ is the rate constant, ${q}_{t}$ (mg g^−1^) is the adsorption capacity at any time t.

The intra-particle diffusion kinetic model assumes that film diffusion has no role, and the adsorption is due to the diffusion of intra-particle only^49,51^. The intra-particle diffusion model is written as Eq. (9):

9 $${q}_{t}={k}_{diff}{t}^{0.5}+C$$

The parameters for thermodynamics for adsorption isotherm can be obtained from isotherms that are dependent on temperature from Eqs. (10)–(12):

10 $${k}_{d}=\frac{{q}_{e}}{{c}_{e}}$$

11 $$\Delta {G}^{0}=-RT\mathit{ln}{k}_{d}$$

12 $$\mathit{ln}{k}_{d}=-\frac{{\Delta H}^{0}}{RT}+\frac{\Delta {S}^{0}}{R}$$

### Characterization

Powder X-Ray Diffraction (PXRD) was conducted by following Bragg’s law of diffraction using RIGAKU make with model SUPERMINI FLEX 6G BENCHTOP facilities from Japan, which was installed at IIT Madras, India. Cu detector D/tex ultra 2 high-speed silicon strip detectors were utilized, covering 2θ ranges from − 3° to + 145°, and the scan speed ranged from 0.01 to 100° per minute. The functional groups of organic and inorganic compounds within the aerogel were identified using IC-Agilent Cary 630 FTIR analyzer from India, which is part of the facilities at IIT Madras, India. The spectrometer used was an ATR-FTIR, operating in the range of 650–4000 cm^−1^. A surface characterization study was conducted using Brunauer–Emmett–Teller (BET) analyzer to determine the pore volume, specific surface area, and pore radius of the adsorbent. This study involved low pressure N_2_ adsorption–desorption isotherms study within the pressure range of 0 to 1 bar and was carried out using JWGB Micro 122W facilities from China, which was also installed at IIT Madras, India. The bath temperature during the study was maintained at 77 K. A morphology study was performed using Hitachi S-4800, HR-SEM equipment from Japan, available at IIT Madras, India. Energy-dispersive X-ray spectroscopy (EDX) was employed to analyze the chemical composition of the aerogel using the same facility. Finally, thermogravimetric analysis (TGA) for the determination of thermal stability of the aerogel was conducted using TA make, SDT-Q600 model TGA analyzer, which is part of the facilities at IIT Madras, India, and is from the USA.

### Experimental theory

The statistical tool Minitab was used to optimize and validate the parameters. Response surface methodology^60^ (RSM) design in the form of central composite design (CCD) was used^28,49,51,61,62^. The variables significant for the adsorption study examined were the pH value of the solution, contaminant concentration, adsorbent dose, and contact time, regarded as the independent factors in randomized trials for CCD. The chosen range of the contaminants in a typical scenario used was a contaminants concentration of 25–70 mg L^−1^, pH of the solution from 4 to 9, contact time of 15–40 min (based on trial experiments), and adsorbent dose of 5–12.5 g L^−1^. The experimental run number was computed in the CCD model using Eq. (13).

13 $$N={2}^{k}+2k+{x}_{o}$$

In this equation, N is the experimental run number, k is the number of factors, and $$x$$_o_ the center point number; the center points are used to estimate pure error for the lack of fit test. Therefore, the experimental run number was found to be 31 ($$k=4, {x}_{0}=7)$$. The constraints were implied as shown in Eq. (14) where $${X}_{i}$$ is obtained from $${X}_{0}$$ the center point with $$\delta X$$ step change.

14 $${x}_{i}=\frac{{X}_{i}-{xX}_{0}}{\delta X}$$

The interaction between the dependent (removal efficiency for each contaminant separately) and independent variables was investigated using the second-order polynomial response equation as the data is non-linear near the optimization. A second-order polynomial approximation of experimental findings expression was also employed to link the independent and dependent (removal efficiency) variables, as shown in Eq. (15)

15 $$y={\beta }_{0}+\sum_{i=1}^{k}{\beta }_{i}{x}_{i}+\sum_{n=1}^{k}{\beta }_{ii}{x}_{i}^{2}+\sum_{i=1}^{k}\sum_{i\ne j=1}^{k}{\beta }_{ij}{x}_{ij}+\varepsilon $$

where $y$ is the response, ${\beta }_{0}$ is a model constant ${\beta }_{i}$ is the linear coefficient, ${\beta }_{ii}$ is the quadratic coefficient, ${\beta }_{ij}$ is the interaction coefficient, ${x}_{i}$ and ${x}_{j}$ are the independent variables (contact time, contaminants concentration, adsorbent dose, and pH), $k$ is the number of independent variables, and $\varepsilon$ is the residual term. The coefficients of the model were calculated using the least square methods of the Minitab package. ANOVA is a statistical technique used to test the relationship between categorical and numerical variables. The test generates the P value to ensure that the data is significant. The analysis of variance (ANOVA) was performed to test the experiment's significance and adequacy, and the obtained results were given a very high F-value (much greater than unity) and a very low probability value, indicating that the model obtained was highly significant. Furthermore, the coefficient of determination R^2^ verified the model's fit. ANOVA analysis (p < 0.05) verified the factorial model and a simplified model was generated by deleting the non-significant variables. The significance of the regression coefficients was determined using a half-normal and a Pareto chart. The best condition for maximal contaminants percentage removal was determined.

## Results and discussions

### GO-SA surface characterization

**Figure 4:**
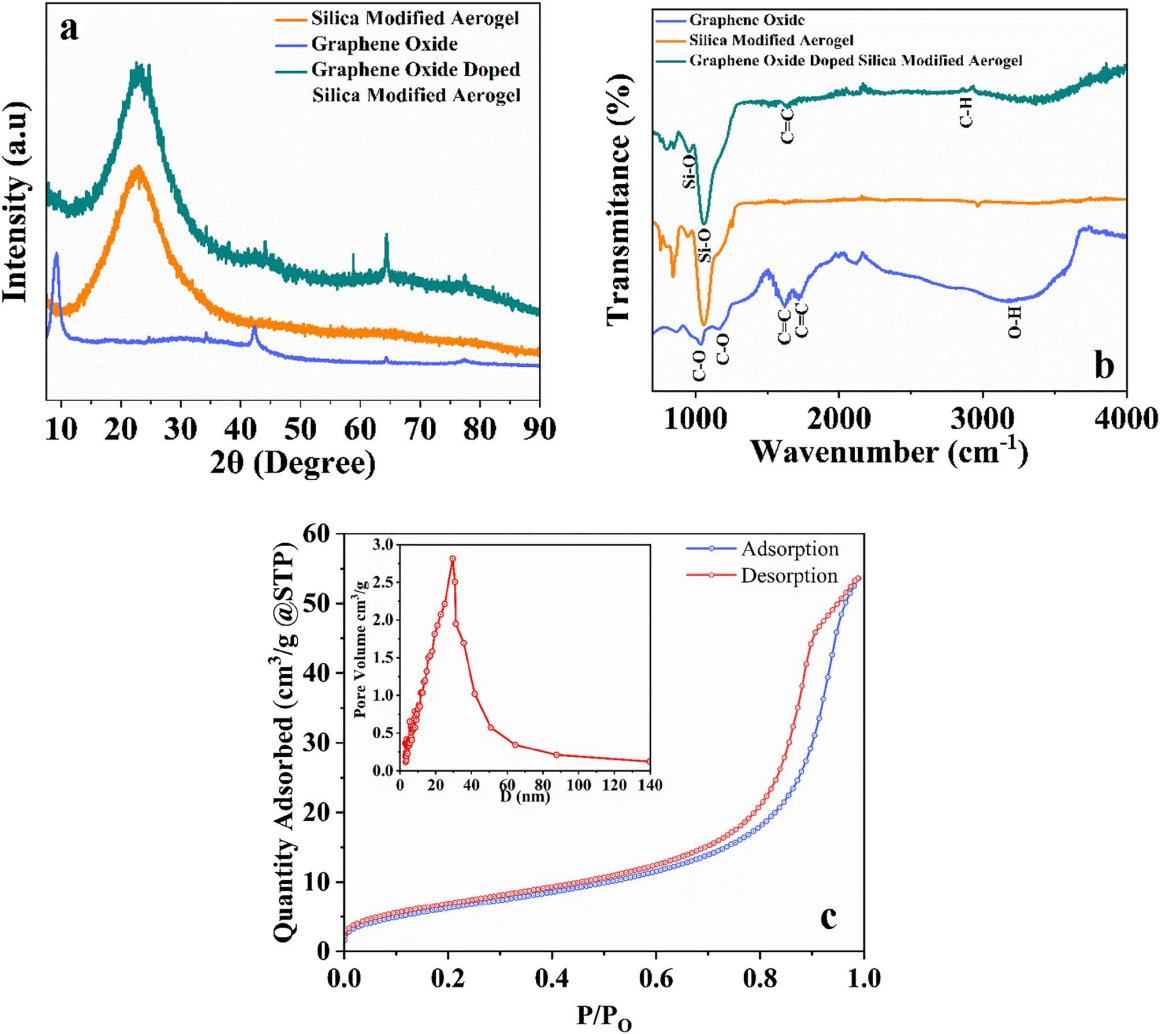
(**a**) XRD Diffractogram of GO-SA, GO, and modified silica aerogel showing the amorphous nature of aerogel, (**b**) FTIR transmittance spectra for GO-SA, GO, and modified silica, (**c**) BET, N_2_ adsorption and desorption isotherm and BJH plot.

The XRD analysis of GO-SA showed a broad peak at 2θ between 20 and 25 degrees, which is associated with the (101) plane of the amorphous silica crystal structure. Graphene oxide typically exhibits a peak at a 2θ value of approximately 10°–12°, corresponding to the (001) plane of the crystal structure. This peak reflects the interlayer spacing of the graphene oxide, which is larger than that of pure graphite due to the presence of oxygen functional groups, as shown in Fig. 4a. These peaks indicate the dominant phase of the aerogel. The FTIR analysis, shown in Fig. 4b, revealed several peaks. For GO-SA a broad peak at around 1100–1200 cm^−1^ was associated with the Si–O–Si stretching vibration of the silica network, while a peak at around 800–900 cm^−1^ was associated with the bending vibration of Si–O–Si^63,64^.

A peak at 1624 cm^−1^ indicated the presence of carbon impurity in the aerogel. A peak at 2966 cm^−1^ also suggested the presence of the C–H group from alkanes^37^. For GO, a broad peak at around 3200–3400 cm^−1^ was associated with the O–H stretching vibrations of the hydroxyl groups, while peaks at around 1700–1750 cm^−1^ and 1050–1250 cm^−1^ were associated with the C=O stretching vibrations of the carboxyl and carbonyl groups, respectively.

**Figure 5:**
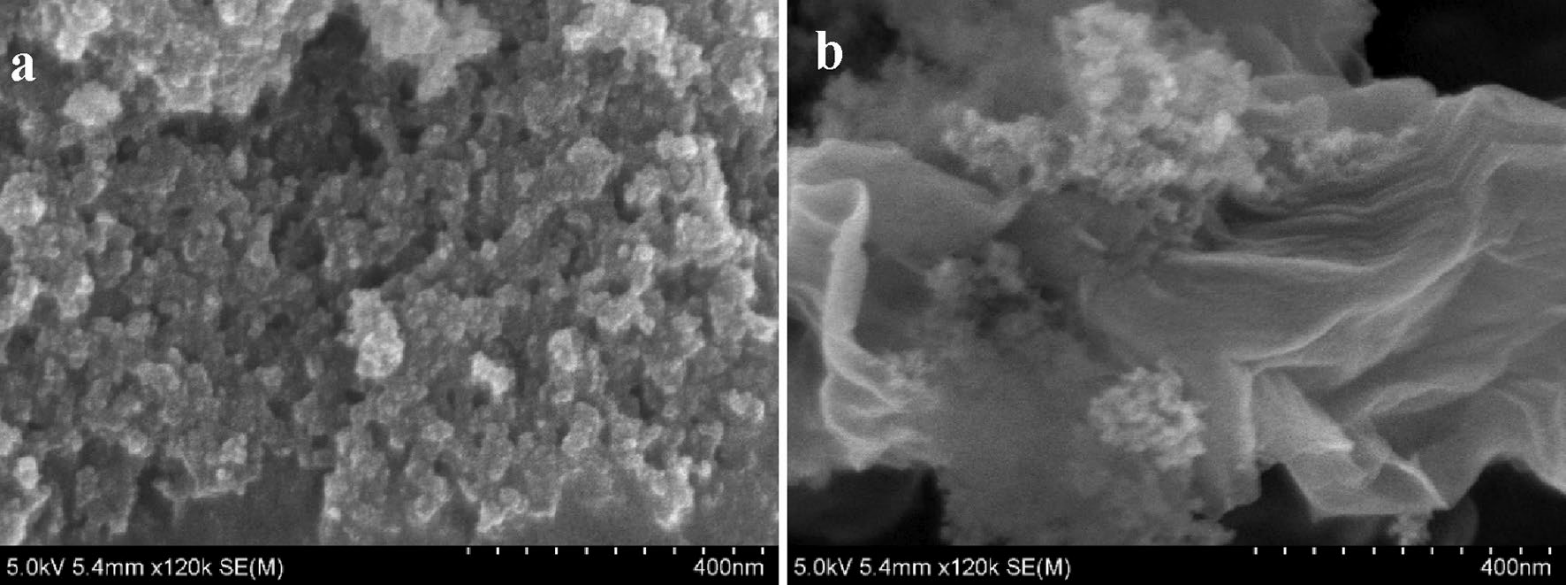
(**a**) A scanning electron micrograph with high resolution has revealed the surface characteristics of GO-SA, displaying its porosity and roughness. (**b**) A high-resolution scanning electron microscope image shows a graphene oxide sheet encompassing the surface of an aerogel.

Figure 4c shows the porosity and surface area of GO-SA aerogel as determined by BET N_2_ adsorption and desorption isotherms^36^. The measured surface area, pore volume, and average pore size were 746.24 m^2^ g^−1^, 2.814 cm^3^ g^−1^, and 29.48 nm, respectively. GO-SA aerogel is mesoporous, as seen in the HR-SEM image showing non-uniform pores in Fig. 5a and b. Desorption on mesoporous aerogel was caused by condensation of the gas by slow extraction^64^. The isotherm of GO-SA aerogel in Fig. 4c shows hysteresis loop type-H3; no limiting adsorption, even at high P/P_0_. It could be said that loose assembly of particles gives rise to pores-like slits that are non-uniform in shape and size^28^. The adsorption graph shows a loop of hysteresis at a high-pressure range (closely P/P_0_ > 0.6), although the desorption line is completely overlapped at the low range pressure (P/P_0_ < 0.4). Suggesting the pores are of ink bottle type, having large pore sizes as the hysteresis occurred at comparatively higher pressures^65^. The existence of prominent mesopores in the aerogel structure was shown by the exceedingly low quantities of nitrogen adsorbed (< 100 cm^3^/g) at relatively low pressures (P/P_0_ > 0.2). This could be said that the adsorption capacity of microporous aerogels was greater than that of mesoporous aerogels. Furthermore, the improved absorption at a relatively high pressure (P/P_0_) range (impending 1.0) established the presence of larger mesopores. The pore size distribution obtained from the BJH method is also shown in Fig. 4c; the BJH model was used to calculate the mesopore size distribution using N_2_ adsorption data; the graph between the pore size and pore volume shows the highest peak at 29 nm, the most frequent diameter. Based on the Kelvin equation, the BJH model^43^ connects the adsorbed/desorbed volume changes at a given pressure to the pore radius that fills/empties at the same pressure. The pores are assumed to be cylindrical, with the Kelvin radius equal to the mesopore radius minus the thickness of the adsorbed layer.

The HR-SEM image Fig. 5a and b characterizes the surface morphology of the GO-SA aerogel, which had distinct particles, a rough surface, and an assorted porous structure, GO biding around the surface. Because of the porous structure, the contaminant molecules were more likely to be adsorbed on the GO-SA aerogel surface by being trapped due to the electrostatic attraction. In addition, EDX in Fig. S1a and b of supplementary information measurements of GO-SA aerogel shows the spread of components on the material's surface. According to the energy dispersive X-ray micrograph, the majority of the GO-SA aerogel consisted of Al, Si, C, and O compounds, which were suited for the adsorption of different contaminants from wastewater. Along with the majority of elements, Au and Mg traces were present due to the sputtering and wafers.

### Parameters optimization for the different process

A total of 31 tests, as per the CCD model, were conducted to optimize the removal percentage of contaminants from synthesized samples. Four different variables—adsorbent dose (AD), contact time (CT), pH, and contaminant concentration (CC)—were tested, and the results are tabulated in supplementary information Table S2. The Percentage normal plot and Pareto chart indicated that the adsorbent dose (AD) and contact time (CT) had the most significant impact on the removal efficiency of contaminants, as their standardized effect was greater than 20, higher than all other factors in Fig. S2 of supplementary information. ANOVA modelling using experimental data produced a Model F-value in Table 1, indicating that the model is significant, and AD, CT, pH, and BC are important model terms. The Lack of Fit F-value in Table 1 showed that the Lack of Fit was insignificant compared to the pure error, as the results had a very high F-value (much greater than unity) and a very low probability value, indicating that the model obtained was highly significant^61^.

**Table 1:** ANOVA (analysis of variance) for quadratic models of response surface.

Contaminants	Source	Degree of freedom	Sum of squares	F-value	P-value	R^2^	Adjusted R^2^
Acid green 25	Model	14	4976.88	95.59	0.00	98.82%	97.78%
	Lack of fit	10	5.93				
	Total	30	5036.38				
Crystal violet	Model	14	5160.77	81.96	0.00	98.62%	97.42%
	Lack of fit	10	7.20				
	Total	30	5232.73				
Sulfamethoxazole	Model	14	4927.02	76.27	0.00	98.52%	97.23%
	Lack of fit	10	7.38				
	Total	30	5000.85				

The ANOVA findings showed a good correlation between the predicted and experimental data. The model successfully navigated the design space described by CCD. Good correlation coefficients (R^2^) of 0.9842 for AG, 0.9862 for CV, and 0.9852 for SMA were observed between the anticipated and experimental effectiveness, suggesting that the model would be accurate in forecasting removal efficiency^22,28,51^. Moreover, the adjusted R^2^ value, which considers the data collection and number of factors in the statistical expressions, was dependent on the degree of freedom. When evaluating the quality of an applied model in a system with multiple independent factors, adjusted R^2^ is preferred. Our study's adjusted R2 values of 0.9778 for AG, 0.9742 for CV, and 0.9723 for SMA were comparable to the respective R^2^ values, indicating a strong match between anticipated and practical removal efficiency. A decreased adjusted R^2^ was obtained due to the existence of multiple mechanisms in the applied model and limited sample size. The acquired F-value and P-value were used for further analysis of the model's relevance^28,49^. In this study, the value of the determination, the coefficient, indicated that the model could explain the variability in the response. Also, the adjusted determination coefficient was high, indicating that the obtained model was significant. To examine the significance of each coefficient and its interactive effects, the corresponding p-values were used as a reference. In the output below, the predictor variables of AG, CV, and SMA are significantly affected because both of their p-values are 0 and less than 0.05. The computed F-values for AG, CV, and SMA were 95.59, 81.96, and 76.75. The resulting F-value for all the contaminants was significantly greater than Fisher's F-value (2.37 at 95% confidence level), showing the model's suitability and competence in describing the sorption of contaminants onto GO-SA. Furthermore, the examination of residuals could be regarded as an acceptable approach for demonstrating how well the model meets the ANOVA hypotheses.

It was found that the contaminant's adsorption was highly dependent on the adsorbent dose and contact time for all the contaminants. To investigate the interaction influence of the operational factors on the adsorption of contaminants, three-dimensional (3D) and contour plots were created based on the polynomial function model in Fig. S3 of Supplementary Information. Response surface methodology may be thought of as a method of predicting removal efficiency for various values of different variables. Contour plots are also useful for identifying the types of interactions between the variables being used. The contour plot shows the possible relationship between three variables. Contour plots show the 3-D relationship in 2-D dimensions; for example, for AG, the contour and response surface plots optimum are very near to adsorbent dose 1 g, contact time 40 min, and removal efficiency more than 90%. The contour plot notes that adsorption is slightly more sensitive to adsorbent dose changes than contact time changes.

The plots between the experimental and predicted results are represented in Fig. S4 of Supplementary Information, indicating that most of the points are near or aligned with the line of regression, showing RSM modelling precision. Moreover, from the residual plots, the predicted results and run number show the error distribution. As a result, the proposed RSM model has been shown to be a useful tool for the prediction of the contaminant's adsorption removal efficiency utilizing GO-SA. The Quadratic equation obtained from the coefficients for removal efficiency is from Eqs. (16)–(18) for different contaminants. Error term behavior is normal, which is confirmed by the straight-line points data.

Removal efficiency (%) for AG with pH, CT (contact time), AD (adsorbent dosage), and CC (contaminant concentration):

16 $$ = { 97}.{37 } + { 1}.{9}0{\text{pH }} + { 3}.{\text{82CT }} + { 9}.{\text{66AD }} - { 2}.{\text{53CC }} - { 1}.{\text{99pH}} \times {\text{CT }} - { 5}.{\text{61pH}} \times {\text{AD }} - { 6}.{\text{43pH}} \times {\text{CC }} + \, 0.{\text{52CT}} \times {\text{AD }} - \, 0.{\text{25CT}} \times {\text{CC }} - \, 0.{\text{69AD}} \times {\text{CC }} - \, 0.{\text{83pH}}^{{2}} + { 1}.{\text{17CT}}^{{2}} + { 1}.{6}0{\text{AD}}^{{2}} + { 2}.0{\text{6CC}}^{{2}} $$

Removal efficiency (%) for CV AG with pH, CT (contact time), AD (adsorbent dosage), and CC (contaminant concentration):

17 $$ = { 98}.{75 } + { 2}.0{\text{6pH }} + { 4}.00{\text{CT }} + { 9}.{\text{51AD }} - { 2}.{\text{34CC }} - { 2}.{\text{31pH}} \times {\text{CT }} - { 5}.{\text{92pH}} \times {\text{AD }} - { 6}.{\text{85pH}} \times {\text{CC }} + \, 0.{\text{26CT}} \times {\text{AD }} - \, 0.{\text{37CT}} \times {\text{CC }} - \, 0.{\text{45AD}} \times {\text{CC }} - \, 0.{\text{67pH}}^{{2}} + \, 0.{\text{98CT}}^{{2}} + { 1}.{\text{46AD}}^{{2}} + { 2}.{\text{27CC}}^{{2}} $$

Removal efficiency (%) for SMA AG with pH, CT (contact time), AD (adsorbent dosage), and CC (contaminant concentration):

18 $$ = { 93}.{87 } + { 1}.{\text{98pH }} + { 3}.{\text{88CT }} + { 9}.{5}0{\text{AD }} - { 2}.{\text{38CC }} - { 2}.0{\text{4pH}} \times {\text{CT }} - { 5}.{\text{68pH}} \times {\text{AD }} - { 6}.{6}0{\text{pH}} \times {\text{CC }} + \, 0.{\text{14CT}} \times {\text{AD }} - \, 0.{\text{49CT}} \times {\text{CC }} - \, 0.{\text{48AD}} \times {\text{CC }} - \, 0.{\text{53pH}}^{{2}} + \, 0.{\text{74CT}}^{{2}} + { 1}.{\text{51AD}}^{{2}} + { 2}.{\text{17CC}}^{{2}} $$

### Removal of contaminants from the water

#### Adsorption mechanism for the contaminants

**Figure 6:**
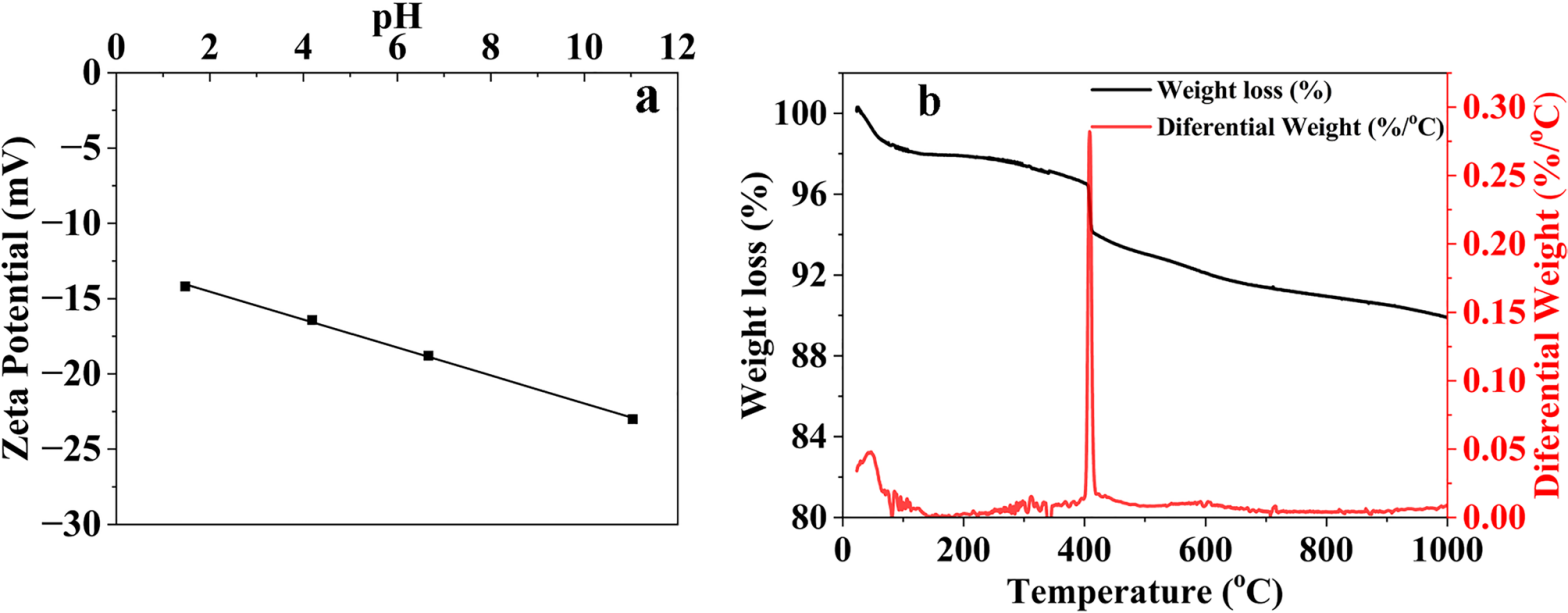
(**a**) The zeta potential plot of the GO-SA at different pH values, (**b**) TGA plot of the mass loss of GO-SA.

The GO-SA aerogel has demonstrated satisfactory potential for removing various contaminants, including AG, CV, and SMA, as shown in this study. Figure 6a displays the zeta potential of GO-SA, which is negative due to the presence of silanol groups (–Si–OH) covering its surface. These groups are partially ionized in aqueous solutions, giving them a net negative charge. The negative charges on the particle's surface repel each other, creating a region of negative charge around the particle and forming the electrical double layer. AG, CV, and SMA.

adsorption onto the GO-SA adsorbent occurs through physical adsorption, as the adsorbate molecules are held on the adsorbent's surface through weak intermolecular forces such as van der Waals or London dispersion forces. The process begins with a rapid dispersion of particles from the solution to the adsorbent, followed by gradual particle diffusion due to mass transfer resistance. As the contaminants in the adsorbent and the liquid phase experience greater repulsion forces, the particle diffusion further slows down. Finally, the available active sites become saturated, marking the end of the process.

Both cationic and anionic dyes exhibit a similar trend, indicating that the adsorbent is pH independent. Ionic interaction was minimal for the contaminants' adsorption on the GO-SA aerogel. These forces are relatively independent of pH, and changes in pH do not significantly affect the strength of the adsorption bond^40,66^. The porosity of the material facilitates pore diffusion, and GO-SA is highly hydrophobic due to the trimethylsilyloxy coating on its surface, which helps with hydrophobic-hydrophobic interaction. The coating has a significant role in the adsorption, as demonstrated in previous studies^37^. The thermal stability of the material was studied using TGA, which showed that the material was highly stable at high temperatures, with only 2% weight loss, as shown in Fig. 6b. This weight loss results from the calcination of the coating, which makes the material hydrophobic after calcination. Experiments were conducted with GO-SA after heating it in a muffle furnace at 600 °C for 1 h in the presence of air to replicate the calcination that occurred in TGA. The adsorption capacity of the material was found to decrease by 60% after heating.

#### Optimizing various parameters of GO-SA aerogel

The optimized parameters obtained from the RSM modelling for GO-SA aerogel were used with minor changes. pH was found to have a minimal role in contaminant adsorption on the aerogel. The Pareto chart indicated that the adsorbent dose played a significant role in removing contaminants. For the adsorption isotherms and kinetic study, we selected neutral pH (i.e., 7), an adsorbent dose of 1.25 g (12.5 g L^−1^) for 100 mL samples, a contact time of 1 h to achieve complete equilibrium, and a contaminant concentration of 100 mg L^−1^ (ppm) after 40 min of contact time between the adsorbent and contaminants at pH 6.5 and room temperature. The maximum removal efficiencies obtained in experiments with maximum contaminant concentration were AG—98.56%, CV—98.64%, and SMA—94.01%.

### Adsorption isotherm

**Figure 7:**
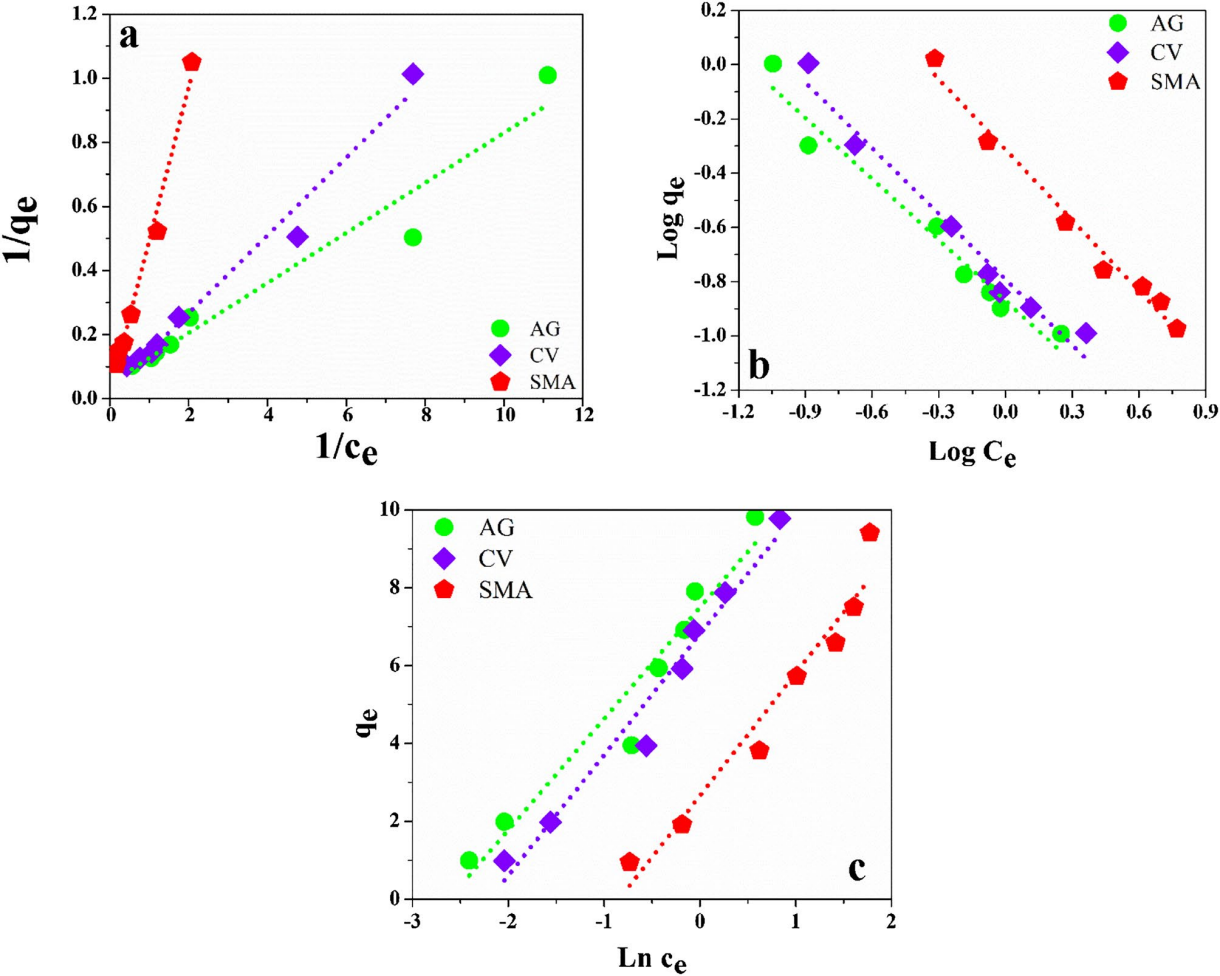
Isotherm plots on GO-SA aerogel at neutral pH, room temperature, and for 40 min (**a**) Langmuir isotherm, (**b**) Freundlich isotherm, (**c**) Temkin Isotherm.

We examined the Temkin, Langmuir, and Freundlich adsorption isotherms on the GO-SA aerogel to further understand the adsorption mechanisms, as shown in Fig. 7a–c and Table 2. We calculated comparative parameters for the adsorption isotherms, including Langmuir, Freundlich, and Temkin, obtained from the linearised plot's slope and an intercept^67^. The correlation coefficient was found to be close to one (R^2^ > 0.95) for all the contaminants. Langmuir and Freundlich's models better fit the experimental data than the Temkin model isotherm, as shown in Table 2, but Langmuir fits the best. The Langmuir model had a higher correlation coefficient value for SMA (R^2^ > 0.99) than the Freundlich model (R^2^ > 0.96), for CV (R^2^ > 0.98) than the Freundlich model (R^2^ > 0.96) while for AG (R^2^ > 0.97) than the Freundlich model (R^2^ > 0.96) indicating that the Langmuir model provides a better explanation of the adsorption onto GO-SA aerogel. The R_L_ separation factor, which is a dimensionless constant that expresses the important features of the Langmuir isotherm, was found to be between 0 and 1, indicating a favorable shape of the isotherm. Indicating the adsorption is favorable for the Langmuir model of the isotherm. The maximum capacity of monolayer exposure q_max_ was found to be 20.56 mg g^−1^, 41.46 mg g^−1^, and 67.07 mg g^−1^ for AG, CV, and SMA, respectively, at room temperature, From Table 2, it can be concluded that the adsorption capacity of GO-SA for dyes and antibiotics is exceptionally high, demonstrating a marked superiority over the performance of other adsorbents, as indicated in Table 3. This robust adsorption capability underscores the pressing need for developing and utilizing materials such as aerogels in water purification applications. The significance of GO-SA's outstanding adsorption capacity lies in its effectiveness, potential cost-effectiveness, and environmental sustainability. In a world grappling with water pollution challenges, GO-SA emerges as a promising solution. Its cost-effectiveness stems from several factors, including the relatively low production costs of graphene oxide (GO) and silica aerogels and the efficient adsorption properties of the composite material. This suggests that GO-SA could offer a more economical option for water purification, especially when compared to alternative adsorbents listed in Table 3 that may exhibit higher adsorption capacities but are likely to come with higher production costs and environmental concerns. Furthermore, the environmental sustainability of GO-SA adds to its appeal. Unlike some adsorbents, which may involve resource-intensive or environmentally detrimental production processes, GO-SA synthesis can align with eco-friendly principles. The combination of graphene oxide with silica aerogels generally involves less resource consumption and can be designed to be environmentally benign and easily scalable production. Therefore, GO-SA holds the potential to contribute positively to the sustainability goals of water purification processes. The physisorption was strongly enhanced for modified silica aerogel due to the supplemental presence of the graphene oxide.

**Table 2:** GO-SA aerogel adsorption isotherm comparative parameters.

Adsorption isotherms	Parameters	AG	CV	SMA
Langmuir	q_max_ (mg g^−1^)	20.56	41.46	67.07
	k_L_ (L mg^−1^)	0.62	0.20	0.03
	R_L_	0.022	0.067	0.314
	R^2^	0.967	0.975	0.987
Freundlich	1/n	− 0.75	− 0.81	− 0.87
	k_f_ (mg g^−1^)	0.14	0.16	0.49
	R^2^	0.958	0.961	0.960
Temkin	k_T_ (L g^−1^)	1.001	1.001	1.000
	B (kJ mol^−1^)	8.744	10.87	8.48
	R^2^	0.934	0.963	0.941

**Table 3:** Adsorption capacity of GO-SA for dye and antibiotics in comparison with other materials.

Adsorbent	Adsorbate	Maximum adsorption capacity (mg g^−1^)	Reference
Silica aerogel	Methylene blue	6.7	^68^
PCS	RTGN	11.2	^69^
MIP-MBC	Sulfamethoxazole	25.65	^70^
Silica gel from rice husk ash	Congo red	25–30	^71^
CS@CNC‒HTA beads	Sulfonamide group	37.62	^72^
ZNO-NP-AC	Green dye	39.1	^73^
Cellulose hydrogel	Direct blue 86	53.76	^74^
Titania-doped silica aerogel	CV	159.89	^75^
Mandarin biochar-TETA	Acid yellow 11	384.62	^76^
Mandarin-biochar-O_3_-TETA	Acid red 35	476.19	^77^
PPAC	Acid yellow 11	515.46	^78^
Modified silica aerogel	AG	15.28	This work
Modified silica aerogel	CV	30.29	This work
Modified silica aerogel	SMA	45.88	This work
GO-SA aerogel	AG	20.56	This work
GO-SA aerogel	CV	41.46	This work
GO-SA aerogel	SMA	67.07	This work

**Table 4:** GO-SA aerogel adsorption kinetics parameters for a different model.

Adsorption kinetics	Parameters	AG	CV	SMA
Pseudo-first-order	q_e_ (mg g^−1^) Cal	5.06	4.87	5.49
	q_e_ (mg g^−1^) Exp	7.15	7.32	8.62
	k_1_ (min^−1^)	0.001	0.001	0.001
	R^2^	0.87	0.85	0.89
Pseudo-second-order	q_e_ (mg g^−1^) Cal	7.30	7.12	9.01
	q_e_ (mg g^−1^) Exp	7.15	7.32	8.62
	k_2_ (g mg^−1^ min^−1^)	0.013	0.015	0.007
	R^2^	0.98	0.98	0.97
Intraparticle diffusion	k_diff_ (mg g^−1^ min^−1^)	1.051	1.028	1.066
	R^2^	0.93	0.92	0.95

### Adsorption kinetics

**Figure 8:**
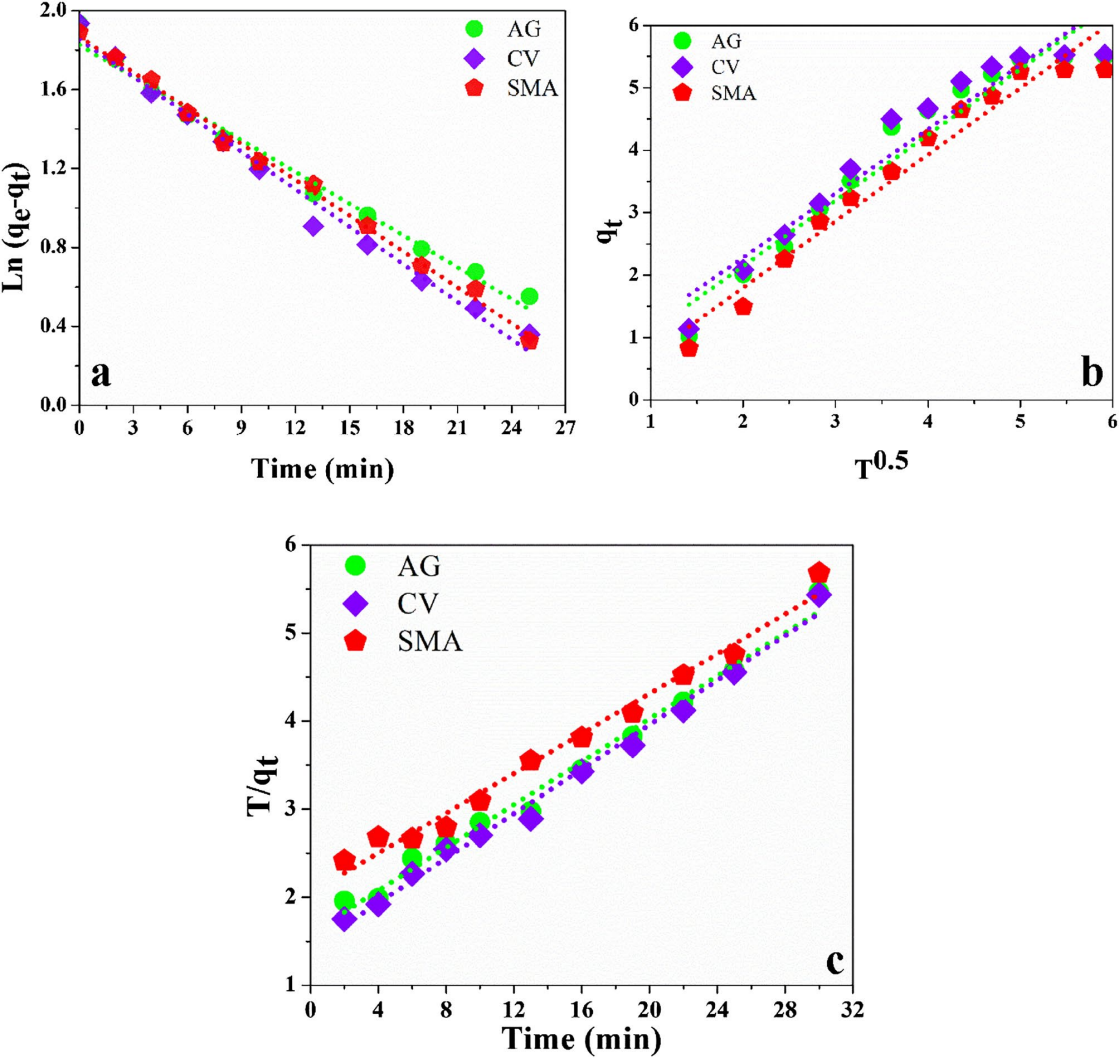
Contaminants adsorption Kinetic models on GO-SA aerogel (**a**) Pseudo-first-order, (**b**) Intra-particle diffusion model, (**c**) Pseudo-second-order.

Three standard kinetic models were used to study the adsorption kinetics of contaminants onto GO-SA aerogel: the pseudo-first-order model, the intraparticle diffusion model, and the pseudo-second-order model, as shown in Fig. 8a and c. Table 4 shows the different parameter values obtained for these models from the linearized equations. The values of R^2^ for the different fitted models for the contaminants indicate that the pseudo-second-order model fits the best. The adsorption capacity of the calculated and experimental values was also similar, further supporting the pseudo-second-order model's application for removing contaminants by adsorption on GO-SA aerogel. The amount of contaminants adsorbed at equilibrium (7.15, 7.32, and 8.62 mg g^−1^ for AG, CV, and SMA, respectively) was similar to the value observed from calculations using the linearized plots of the pseudo-second-order model. The adsorption process was fast in the initial 20 min due to the large availability of pores and adsorption surface but slowed down over time due to saturation of the adsorbent's surface area and slow diffusion into its micropores. Although the other two kinetic models (the pseudo-first-order and intraparticle diffusion models) were not proficient, the pseudo-second-order kinetics were the most accurate, with the highest correlation coefficients (R^2^ = 0.99).

### Adsorption thermodynamic

**Figure 9:**
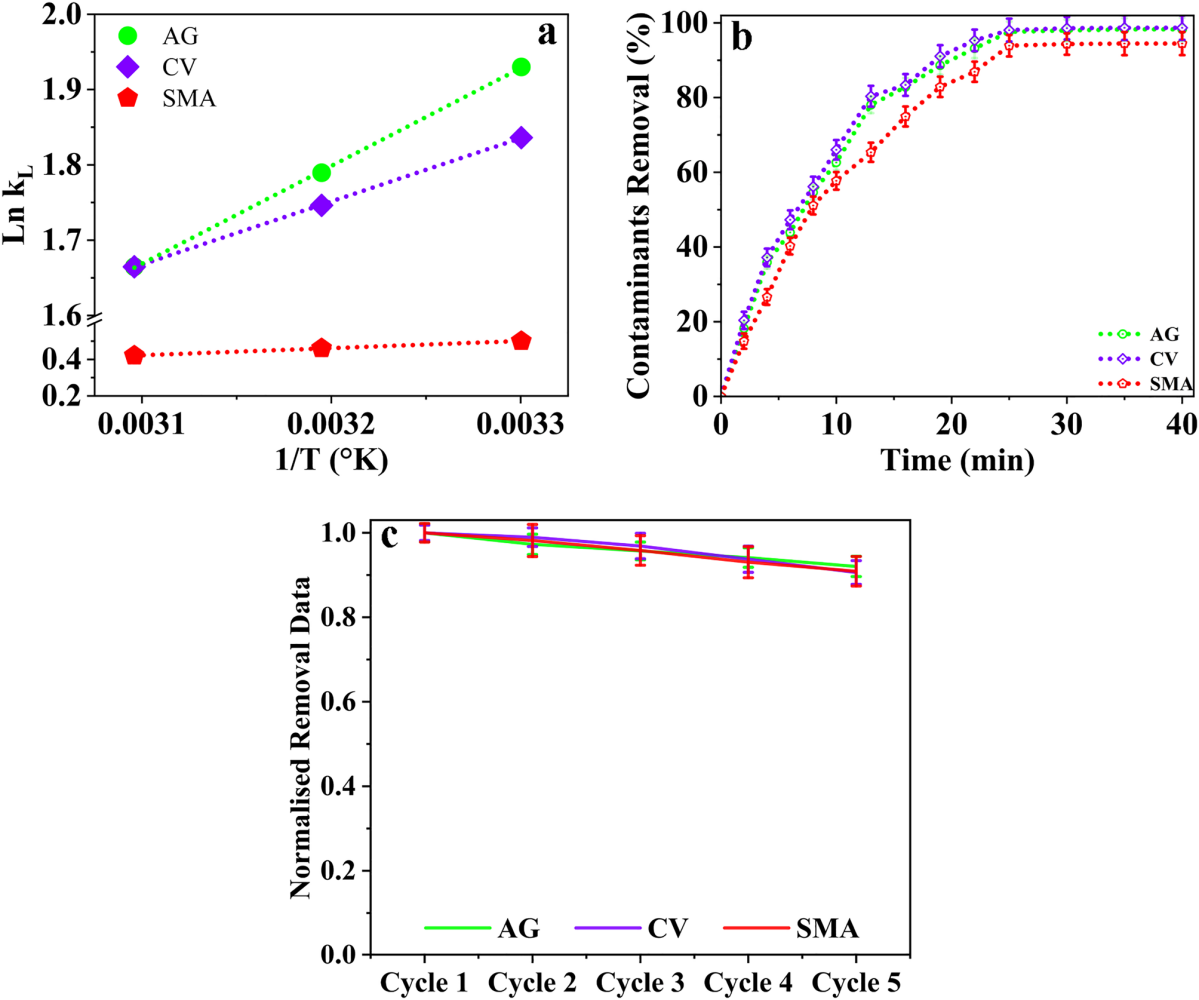
(**a**) GO-SA aerogel adsorption thermodynamic, (**b**) removal efficiency, (**c**) Regeneration and reusability of GO-SA aerogel for removal of contaminants.

The thermodynamics of adsorption offer insights into the energy changes taking place in the adsorbent material, as well as the mechanism underlying the adsorption process. A ΔH° value exceeding 40 kJ mol^−1^ indicates that the adsorption is a chemisorption process, whereas a value below 40 kJ mol^−1^ indicates physisorption. In this study ΔH° is negative confirming that the adsorption through physisorption. The entropy (ΔS°) and enthalpy (ΔH°) changes are obtained from the linearized plots between Ln k_l_ versus 1/T, as shown in Fig. 9a. The Gibbs free energy ΔG° values were calculated from the equation at 303 K, and the values obtained were negative for all the contaminants ranging from 1 to 5 kJ mol^−1^. The negative ΔG° values, combined with the negative ΔH° values calculated for all the contaminants in Table 5, show that the adsorption process on GO-SA aerogel is exothermic, spontaneous, and feasible^79,80^.

**Table 5:** Adsorption thermodynamics on GO-SA aerogel.

Adsorption thermodynamics	AG	CV	SMA
ΔH° (kJ mol^−1^)	− 10.795	− 6.974	− 3.235
ΔS° (kJ mol^−1^ K^−1^)	− 0.019	− 0.007	− 0.006
ΔG° (kJ mol^−1^) @ 303°K	− 4.862	− 4.625	− 1.261
ΔG° (kJ mol^−1^) @ 313°K	− 4.656	− 4.544	− 1.199
ΔG° (kJ mol^−1^) @ 323°K	− 4.470	− 4.447	− 1.131

The ΔG° value decreased from − 4.862 to − 4.470 kJ mol^−1^ with the increase in temperature from 303 to 323 K for AG on the GO-SA aerogel. The same trend was found with the other contaminants. Therefore, all the experiments were carried out at room temperature. The entropy ΔS° value was negative, which is expected as orderliness increased upon adsorption on GO-SA aerogel. A negative value of ΔS° suggests that the adsorption process decreased the randomness or disorder of the system.

### Selectivity

A mixture of AG and CV at 50 mg L^−1^ each was used to evaluate the selectivity of GO-SA. The experiment was conducted at room temperature and neutral pH with an adsorbent dose of 1.25 g. The results showed that GO-SA had the highest removal percentage, 96.9%, for both dyes. The adsorbent GO-SA showed the same adsorption levels and rates for our anionic and cationic dyes. However, it was found that the material did not exhibit significant selectivity towards either dye. GO-SA has a superhydrophobic surface that interacts with the hydrophobic regions of dye molecules, and its high surface area and porous structure can effectively adsorb a wide range of dyes, including AG and CV. This can result in non-selective adsorption of both CV and AG. The non-selective GO-SA offers a highly efficient, cost-effective, and versatile solution for cationic and anionic dye adsorption.

### Removal efficiency

The removal efficiency of different contaminants, such as dyes and antibiotics, was investigated using GO-SA aerogel under optimized conditions of pH 7 (close to neutral), contact time of 1-h, adsorbent dose of 1.25 g (12.5 g L^−1^), and contaminant concentration of 100 mg L^−1^. The maximum removal efficiency was found to be 98.23%, 98.71%, and 94.46% for acid green 25 (AG), crystal violet (CV), and sulfamethoxazole (SMA), respectively, as shown in Fig. 9b. As the experiments were conducted in triplicate, the maximum error obtained in the removal efficiency was ± 3.5%, which is within an acceptable range. The high removal efficiency can be attributed to the material's highly porous and superhydrophobic nature and structure. The hydrophobicity reduces the direct interaction of water with the adsorbent and ensures better binding between the contaminants and the active sites in the aerogel.

### Reusability and regeneration of GO-SA aerogel

Due to the saturation of pores and active sites by contaminants, the adsorption of contaminants on the GO-SA aerogel slows down, resulting in a decrease in aerogel activity^25,51,67^. The reusability of the GO-SA aerogel was investigated by eluting the contaminants using different eluants. An alkaline eluant of 0.6M acetic acid was used to remove anionic dyes, such as crystal violet, followed by washing with methanol to completely elute the anionic dye. For cationic dyes, such as acid green 25, 10g L^−1^ of peroxydisulfate^81^ was used for three cycles, followed by washing with methanol. A mixture of ethanol, methanol, and acetone in the ratio 35:35:30, respectively, was used as the eluant for sulfamethoxazole antibiotics. The maximum desorption efficiency obtained for all contaminants was between 85 and 90%. After desorption, the same aerogel was used for another cycle of adsorption, and the removal efficiency after each cycle of reuse is shown in Fig. 9c. After each adsorption cycle, the GO-SA aerogel's contaminant removal efficiency decreased by 2–5%. In future work, the regeneration efficiency could be further optimized. However, this study carried out a reusability study for five cycles, and the removal efficiency decreased after each cycle. Nevertheless, even after five cycles, the removal efficiency remained reasonable. The reusability study confirmed that the GO-SA aerogel could be reused multiple times, reducing the cost burden, and having minimal environmental impact.

## Applications of GO-SA aerogel

The adsorption capabilities of GO-SA as an adsorbent were investigated in the context of dye removal. The study involved the use of a sample collected from lake water, characterized by a notably high Total Organic Carbon (TOC) content, approximately 50 mg L^−1^, and a substantial Total Inorganic Carbon (TIC) content, approximately 70 mg L^−1^. In the experimental setup, the water sample from the lake was dosed with a contaminant concentration of 100 mg L^−1^ and was introduced to the GO-SA adsorbent. The primary objective of this investigation was to assess the removal efficiency of the contaminants from the water matrix through the adsorption process facilitated by GO-SA. The results of the study have yielded promising outcomes regarding the removal efficiency of the contaminants with minimum impact on the value of TOC and TIC. Scalability assessment is a pivotal aspect of process engineering, particularly in the context of developing novel adsorption techniques for water purification. In our study, we meticulously evaluated the scalability of the adsorption process using a semi-batch reactor, the design of which is elucidated in Fig. 2b. In this experimental setup, we initiated the investigation with a representative initial contaminant concentration of 100 mg L^−1^. This concentration was deliberately selected as a reference point for most of our study, serving as a consistent baseline for our evaluations. Such standardization facilitates a clear comparison of the adsorption performance across various experimental conditions.

The heart of the scalability assessment lay in the reactor's column, which was characterized by a specific length of 0.5 m. Furthermore, the flow rate employed in this experiment was meticulously controlled at 0.2 L min^−1^. This controlled flow rate was instrumental in achieving a precise retention time of at least 2.5 min within the column. The significance of this controlled retention time lies in its role in facilitating effective adsorption. By allowing the water to remain within the column for this duration, we ensured that the adsorption process could reach its equilibrium state, optimizing contaminant removal. This systematic and scientific approach to scalability assessment is critical in understanding the feasibility and potential of the adsorption process for large-scale water purification applications. It provides valuable insights into how the technology can be applied and expanded to address real-world challenges related to water purification.

The investigation of scalability potential is a crucial aspect of experimental research, and it is being systematically assessed through the implementation of a continuous setup, as illustrated in Fig. 2c. In this experimental configuration, a reactor with a fixed volume of 3 L has been employed and densely packed with aerogel, the adsorbent under examination. To assess the scalability of the adsorption process, an initial contaminant concentration of 100 mg L^−1^ has been selected as the representative condition for these experiments. This specific concentration serves as a standardized baseline, allowing for consistent comparisons across various experimental scenarios.

The flowrate within the reactor has been carefully regulated at 1 L min^−1^. This controlled flowrate ensures that the aqueous solution containing contaminants is continuously introduced into the reactor. It is worth noting that this flowrate provides a retention time for the wastewater of precisely 3 min within the reactor. This duration is a critical parameter as it governs the time available for the adsorption process to occur. The scientific significance of this experimental setup lies in its ability to mimic and assess real-world conditions and demands for scalability. By employing a continuous reactor setup with defined parameters, we gain insights into how the adsorption process behaves under continuous flow conditions, a key consideration for practical applications in water purification. The removal in the semi-batch reactor was > 85%, and in the continuous setup, it was found to be > 76%. Modifying and testing the process and setup to improve the removal percentage for using GO-SA on a large scale is ongoing. GO-SA aerogel can be used in real-life for a sustainable and eco-friendly environment. It can be used as a primary purification for wastewater from the textile industry. In this study, the modified aerogel has shown promising results for the removal of Acid Green 25 (AG), Crystal Violet (CV), and Sulfamethoxazole (SMA), along with other contaminants. This study indicates that GO-SA aerogel can be applied at real-field scales. Still, further work is needed to establish the technology as it shows a promising solution for the adsorption of the contaminants.

## Conclusion

The GO-SA aerogel has shown excellent adsorption capacity for various contaminants. In this study, experiments were performed using Acid Green and Crystal Violet and Sulfamethoxazole, commonly used to treat different bacterial infections. It was observed that the driving force for the separations was a combination of electrostatic attraction and high hydrophobic interaction. Response surface methodology's central composite design was used to optimize parameters such as pH, contact time, adsorbent dose, and contaminant concentration. Our experimental results matched well with the predicted results of RSM. The adsorbent dose and contact time were the dominating factors for the adsorption of contaminants. The adsorption isotherm study showed that the Langmuir isotherm model best fitted the data obtained from the experiments. Separation factors less than 1 and greater than 0 confirmed that the adsorption was favorable, with a maximum adsorption capacity of 20.56, 41.46, and 67.07 mg g^−1^ for Acid Green 25, Crystal Violet, and Sulfamethoxazole, respectively. The kinetic model that best fit this study was the pseudo-second-order; calculated values of equilibrium were 7.30, 7.12, and 9.01 mg g^−1^ for Acid Green 25, Crystal Violet, and Sulfamethoxazole, respectively was very close to experimental values. The adsorption thermodynamics showed that the adsorption process was exothermic and spontaneous. The highly versatile material GO-SA aerogel was found to be an eco-friendly and sustainable adsorbent for the effective removal of contaminants.

### Supplementary Information

Supplementary Information.

## Data Availability

All data generated or analyzed during this study are included in this published article and its supplementary information files.
