# Supplementary material for: Preparation of graphene oxide-doped silica aerogel using supercritical method for efficient removal of emerging pollutants from wastewater
Source: Sci Rep. 2023 Sep 30;13:16448. doi: 10.1038/s41598-023-43613-w (PMC10542781; doi:10.1038/s41598-023-43613-w)
Supplement: Supplementary file 1 — Supplementary Information. [file 41598_2023_43613_MOESM1_ESM.docx]

**Supplementary information**

**Preparation of Graphene Oxide-Doped Silica Aerogel using Supercritical Method for Efficient Removal of Emerging Pollutants from Wastewater**

Subhash Kumar Sharma^1^, P. Ranjani^1^, Hadas Mamane^2^, and Rajnish Kumar^1*^

*^1^Department of Chemical Engineering, Indian Institute of Technology Madras, Chennai-600036, India*

*^2^School of Mechanical Engineering, Faculty of Engineering, Tel Aviv University, Tel Aviv 69978, Israel*

****Corresponding Author:*** *rajnish@iitm.ac.in*

Table.S1 Contaminants Characteristics.

| Name | Chemical structure | Molecular formula | Mw (g/mol) | λ_max_ (nm) | Type |
| --- | --- | --- | --- | --- | --- |
| Acid green 25 | 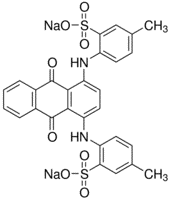 | C_28_H_20_N_2_Na_2_O_8_S_2_ | 622.58 | 625 | Anionic |
| Crystal violet | 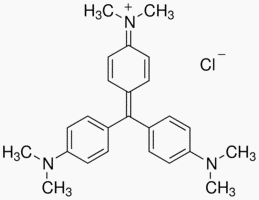 | C_25_H_30_N_3_Cl | 407.98 | 590 | Cationic |
| Sulfamethoxazole | 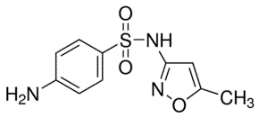 | C_10_H_11_N_3_O_3_S | 253.28 | 254 | Antibiotic |

Table S2. The removal Efficiency value of the four factorial CCD matrix with a predicted value

| Std. Order | Run Order | pH | Contact time  (min) | Adsorbent (g/L) | [contaminants Concentration] _o_ (mg/L) | Removal efficiency (%) | | | | | |
| --- | --- | --- | --- | --- | --- | --- | --- | --- | --- | --- | --- |
|  |  |  |  |  |  | Acid Green 25 | | Crystal Violet | | Sulfamethoxazole | |
|  |  |  |  |  |  | Exp. | Pre. | Exp. | Pre. | Exp. | Pre. |
| 22 | 1 | 6.5 | 27.5 | 16.25 | 47.5 | 91.25 | 90.97 | 89.99 | 90.38 | 87.25 | 86.45 |
| 6 | 2 | 9.0 | 15.0 | 12.5 | 25.0 | 94.36 | 92.89 | 94.27 | 92.64 | 90.15 | 88.81 |
| 12 | 3 | 9.0 | 40.0 | 5 | 70.0 | 74.86 | 75.38 | 75.26 | 75.76 | 71.24 | 71.72 |
| 4 | 4 | 9.0 | 40.0 | 5 | 25.0 | 84.45 | 83.01 | 84.27 | 83.39 | 80.45 | 78.86 |
| 3 | 5 | 4.0 | 40.0 | 5 | 25.0 | 77.58 | 76.66 | 78.56 | 77.78 | 74.58 | 73.88 |
| 18 | 6 | 11.5 | 27.5 | 8.75 | 47.5 | 92.74 | 93.22 | 93.78 | 93.63 | 89.82 | 89.68 |
| 9 | 7 | 4.0 | 15.0 | 5 | 70.0 | 63.12 | 63.34 | 62.79 | 63.12 | 58.62 | 58.58 |
| 28 | 8 | 6.5 | 27.5 | 8.75 | 47.5 | 97.36 | 97.37 | 98.75 | 98.75 | 93.87 | 93.87 |
| 16 | 9 | 9.0 | 40.0 | 12.5 | 70.0 | 98.56 | 99.77 | 98.64 | 100.39 | 94.01 | 95.58 |
| 23 | 10 | 6.5 | 27.5 | 8.75 | 2.5 | 99.89 | 104.53 | 99.46 | 104.47 | 94.36 | 99.16 |
| 1 | 11 | 4.0 | 15.0 | 5 | 25.0 | 75.88 | 74.07 | 76.38 | 73.93 | 72.53 | 69.63 |
| 24 | 12 | 6.5 | 27.5 | 8.75 | 92.5 | 98.12 | 94.40 | 99.01 | 95.11 | 93.12 | 89.66 |
| 30 | 13 | 6.5 | 27.5 | 8.75 | 47.5 | 96.95 | 97.37 | 98.75 | 98.75 | 93.87 | 93.87 |
| 14 | 14 | 9.0 | 15.0 | 12.5 | 70.0 | 86.48 | 87.08 | 87.86 | 88.24 | 83.64 | 84.33 |
| 2 | 15 | 9.0 | 15.0 | 5 | 25.0 | 83.08 | 81.41 | 82.63 | 81.02 | 78.35 | 76.60 |
| 10 | 16 | 9.0 | 15.0 | 5 | 70.0 | 66.45 | 67.36 | 66.11 | 67.55 | 62.57 | 63.43 |
| 25 | 17 | 6.5 | 27.5 | 8.75 | 47.5 | 97.45 | 97.37 | 98.75 | 98.75 | 93.87 | 93.87 |
| 31 | 18 | 6.5 | 27.5 | 8.75 | 47.5 | 97.45 | 97.37 | 98.75 | 98.75 | 93.87 | 93.87 |
| 19 | 19 | 6.5 | 2.5 | 8.75 | 47.5 | 66.12 | 67.27 | 65.48 | 67.06 | 61.72 | 63.41 |
| 21 | 20 | 6.5 | 27.5 | 1.25 | 47.5 | 51.13 | 52.33 | 51.62 | 52.34 | 46.33 | 48.47 |
| 17 | 21 | 1.5 | 27.5 | 8.75 | 47.5 | 85.17 | 85.61 | 84.12 | 85.38 | 80.27 | 81.76 |
| 29 | 22 | 6.5 | 27.5 | 8.75 | 47.5 | 97.45 | 97.37 | 98.75 | 98.75 | 93.87 | 93.87 |
| 7 | 23 | 4.0 | 40.0 | 12.5 | 25.0 | 97.10 | 95.59 | 97.27 | 95.12 | 93.15 | 90.96 |
| 20 | 24 | 6.5 | 52.5 | 8.75 | 47.5 | 82.78 | 82.55 | 83.54 | 83.06 | 79.26 | 78.91 |
| 13 | 25 | 4.0 | 15.0 | 12.5 | 70.0 | 84.98 | 85.82 | 85.43 | 85.60 | 81.13 | 81.39 |
| 26 | 26 | 6.5 | 27.5 | 8.75 | 47.5 | 97.45 | 97.37 | 98.75 | 98.75 | 93.87 | 93.87 |
| 5 | 27 | 4.0 | 15.0 | 12.5 | 25.0 | 89.15 | 88.31 | 88.23 | 87.34 | 84.26 | 83.76 |
| 11 | 28 | 4.0 | 40.0 | 5 | 70.0 | 71.48 | 72.35 | 71.89 | 72.81 | 68.84 | 68.85 |
| 27 | 29 | 6.5 | 27.5 | 8.75 | 47.5 | 97.45 | 97.37 | 98.75 | 98.75 | 93.87 | 93.87 |
| 8 | 30 | 9.0 | 40.0 | 12.5 | 25.0 | 99.70 | 99.16 | 99.67 | 98.94 | 94.01 | 94.03 |
| 15 | 31 | 4.0 | 40.0 | 12.5 | 70.0 | 98.16 | 99.51 | 98.01 | 99.23 | 92.89 | 94.63 |


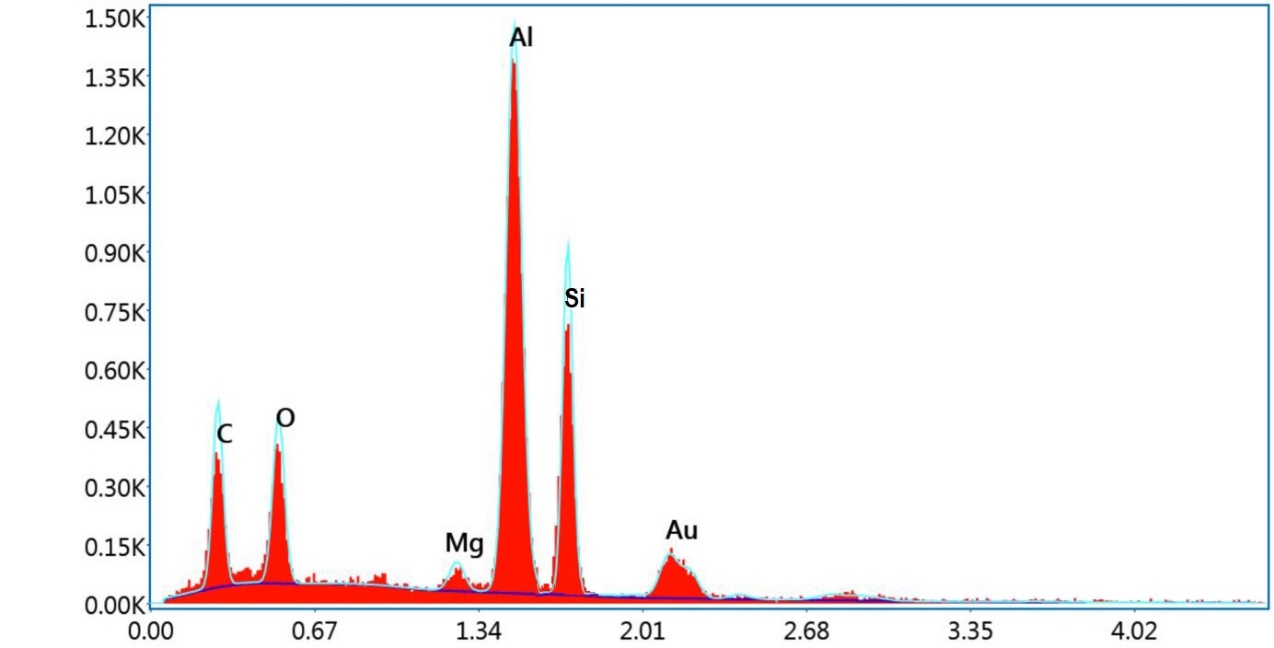


Fig.S1-a: EDX of P400-Aerogel


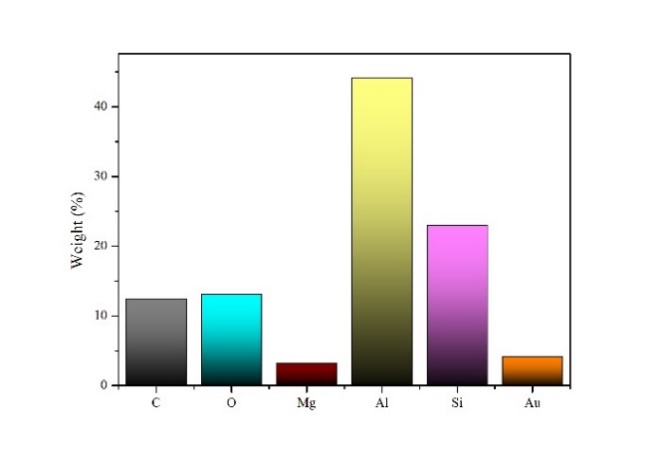


Fig.S1-b: Approx elemental composition of P400-Aerogel


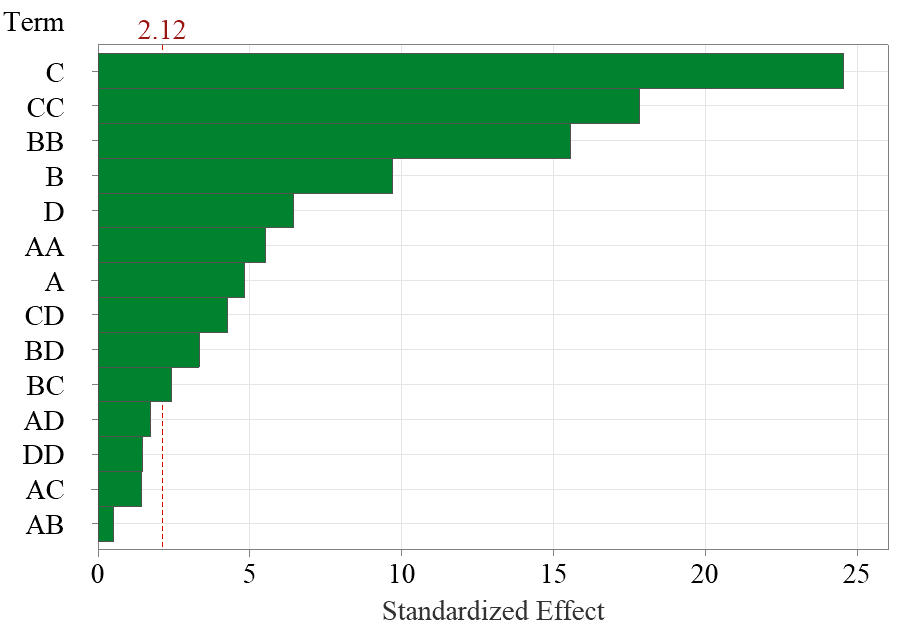

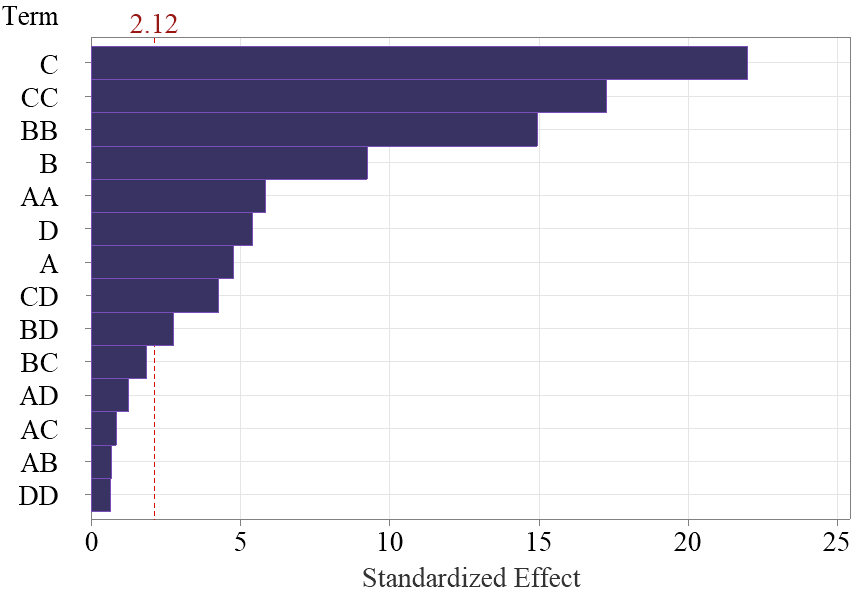

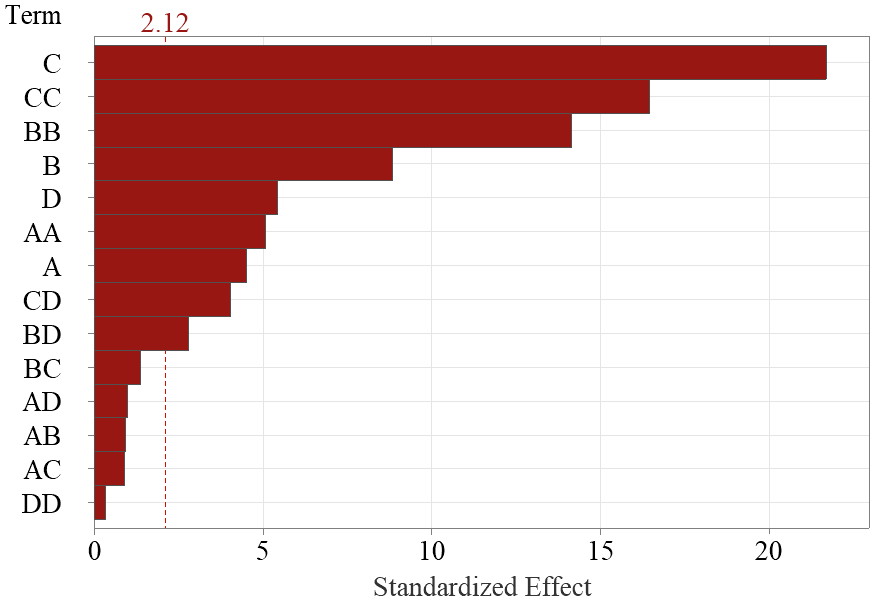


c

b

a

Fig. S2: Pareto Chart of the standardized effects (Removal (%), α=0.05) (a) AG, (b) CV, (c) SMA (Terms A= pH, B= CT, C= AD, D= CC, CC= ADxAD, BB= CTxCT, AA= pHxpH, CD= ADxCC, BD= CTxCC, BC= CTxAD, AD= pHxCC, AB= pHxCT, AC= pHxAD, DD= CCxCC)


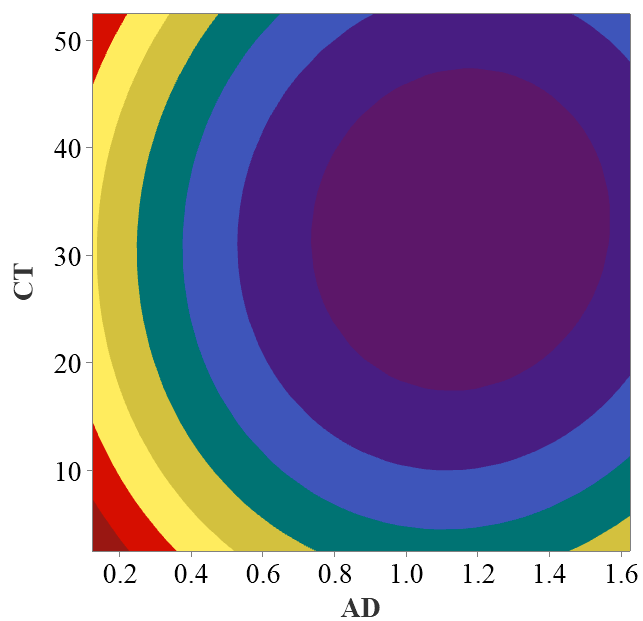

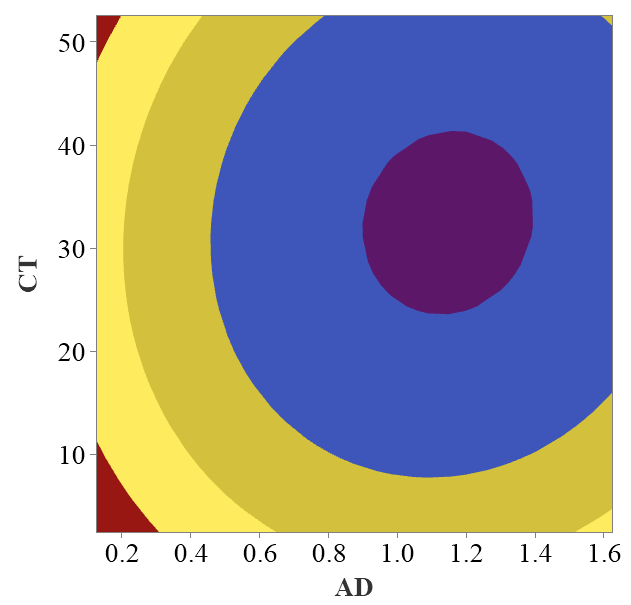

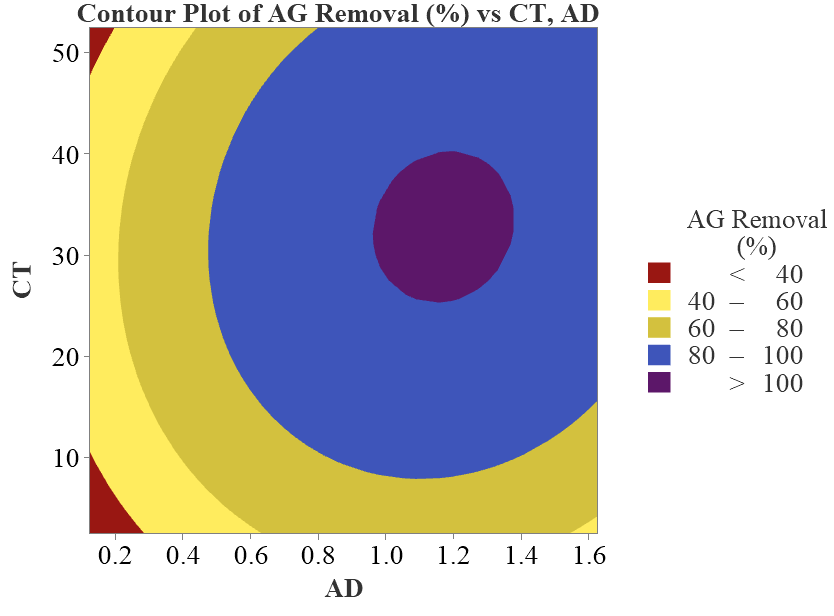


c

b

a


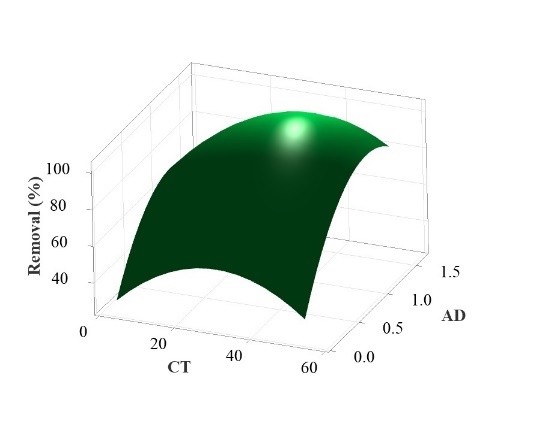

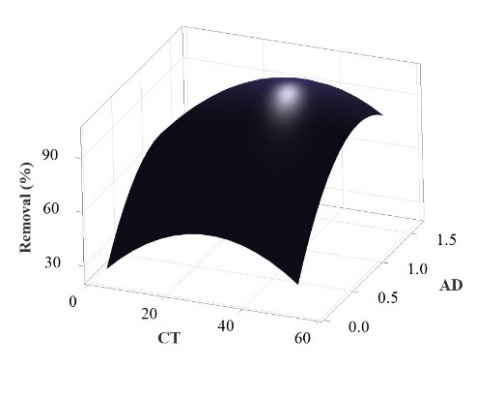

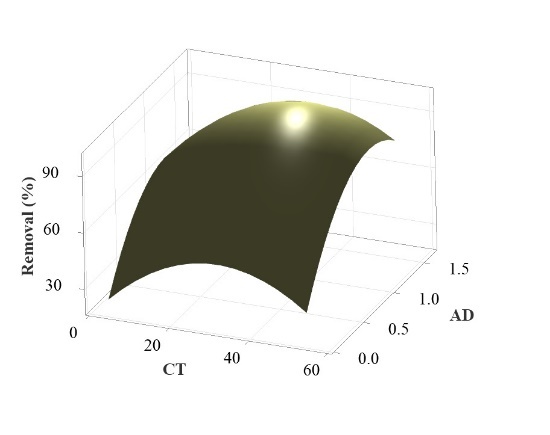


Fig. S3: Contour Plot and surface plots of contaminants removal (%) vs CT, AD (a) AG, (b) and (c) SMA (Contour plot colour represents
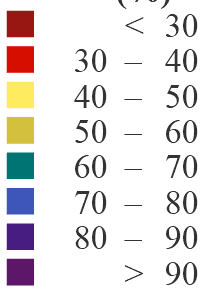

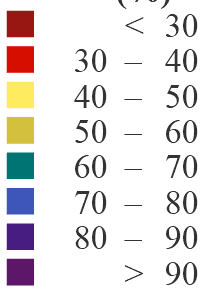

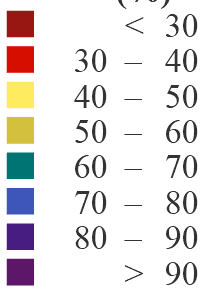

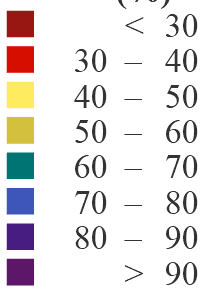

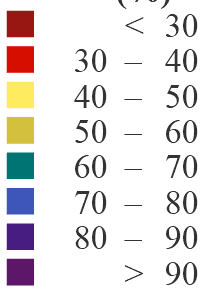

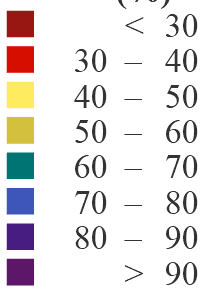

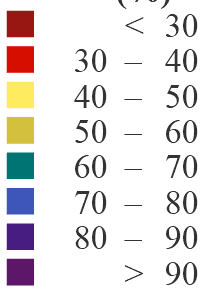

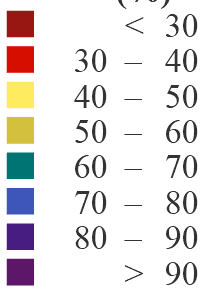
)


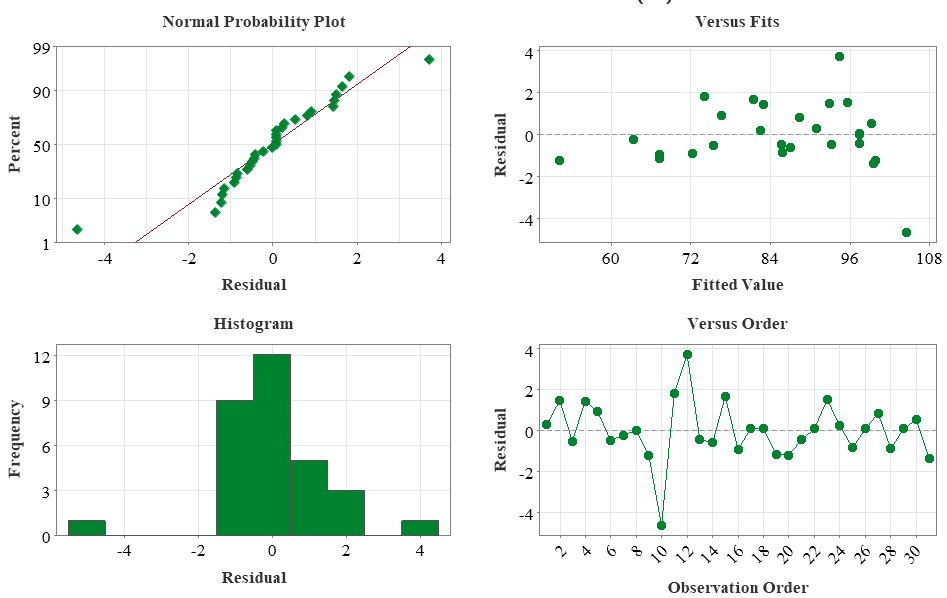

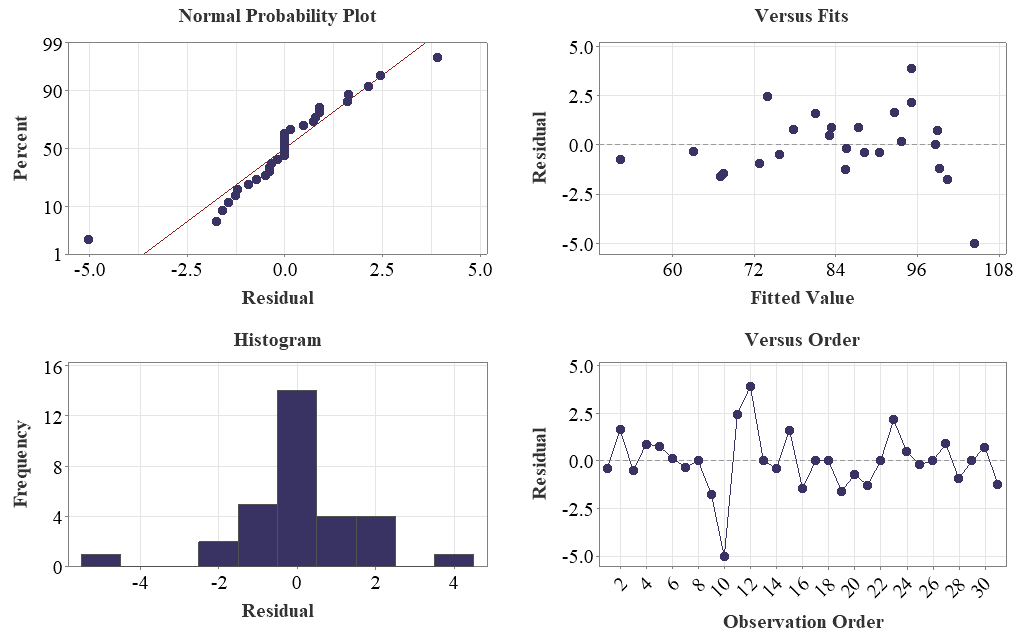

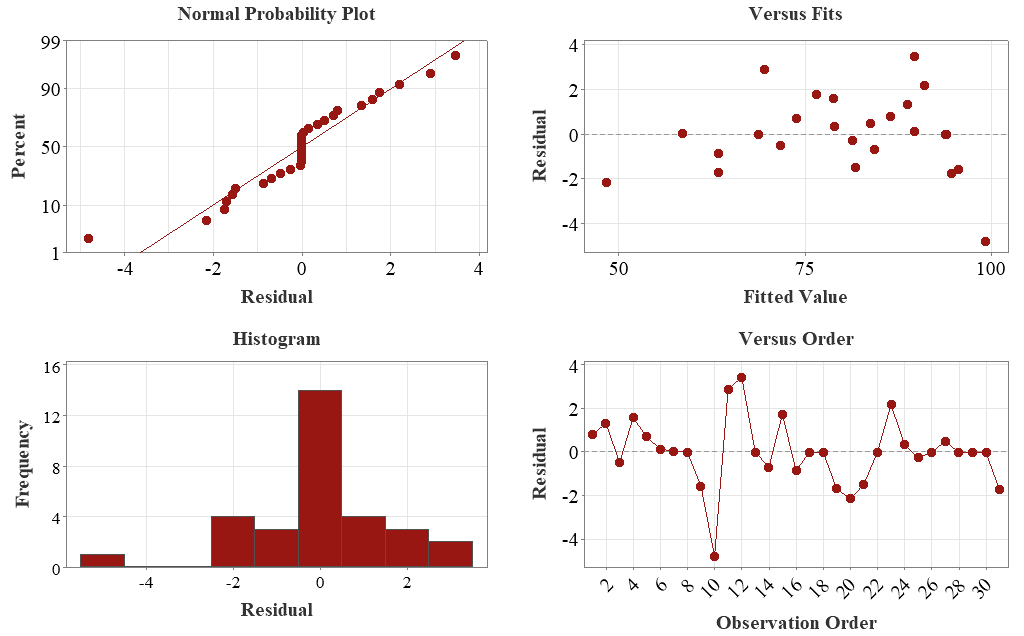


C

b

a

Fig. S4. Residual Plots (a) AG, (b) CV, and (c) SMA
